# Comprehensive analysis of m5C-Related lncRNAs in the prognosis and immune landscape of hepatocellular carcinoma

**DOI:** 10.3389/fgene.2022.990594

**Published:** 2022-10-20

**Authors:** Qian Lu, Lianyu Liu, Shuai Wang, Qi Zhang, Li Li

**Affiliations:** ^1^ Department of Gastroenterology, The Affiliated Hospital of Xuzhou Medical University, Xuzhou, China; ^2^ Institute of Digestive Diseases, Xuzhou Medical University, Xuzhou, China; ^3^ Department of General Surgery, The Affiliated Hospital of Xuzhou Medical University, Xuzhou, China; ^4^ The Graduate School, Xuzhou Medical University, Xuzhou, China

**Keywords:** m5C, lncRNA, hepatocellular carcinoma, prognosis, immune landscape

## Abstract

5-Methyladenosine (m5C) is a type of epigenetic modification involved in the progression of various cancers. To investigate the role of m5C-related long non-coding RNAs (lncRNAs) in the prognosis and immune cell infiltration in hepatocellular carcinoma (HCC), we obtained patients’ clinical information and transcriptome data of HCC from the Cancer Genome Atlas (TCGA) database. We applied Pearson correlation analysis to construct an m5C-related lncRNA–messenger RNA (mRNA) co-expression network. Univariate Cox analysis, least absolute shrinkage and selection operator (LASSO), and multivariate Cox analysis were employed to establish an m5C-related lncRNA prognostic risk model. We then verified the model using Kaplan–Meier analysis, principal component analysis, as well as univariate and multivariate Cox analyses. The expression of m5C-related lncRNAs was validated in HCC tissues and different cell lines. Combining the risk score and clinicopathological features, a nomogram was established for predicting the overall survival (OS) of HCC patients. Furthermore, gene set enrichment analysis (GSEA) revealed that some tumor-associated pathways were significantly enriched in the high-risk group. Immune cell infiltration analysis demonstrated that the levels of Treg cells, neutrophils, and M2 macrophages were higher in the high-risk group. In addition, patients with high tumor mutation burden (TMB) had worse OS than those with low TMB. We also assessed the immune checkpoint level and chemotherapeutic agent sensibility. Then *in vitro* experiments were performed to examine the biological function of MKLN1-AS in HCC cells and found that knockdown of MKLN1-AS suppressed the proliferation, migration, and invasion. In conclusion, m5C-related lncRNAs played a critical role in predicting the prognosis of patients with HCC and may serve as new therapeutic targets for HCC patients.

## Introduction

Hepatocellular carcinoma (HCC) is one of the most common malignancies and the fourth leading cause of cancer-related deaths worldwide (Ma et al., 2016; Xu et al., 2022). Many types of pharmaceutical therapies have been approved to treat HCC, including targeted tyrosine kinase inhibitors, immune-based therapies, and combination of chemotherapy. However, due to chemoresistance and immunosuppressive elements, current therapies have not effectively improved the outcome for HCC patients (Foerster et al., 2022). Therefore, there is an urgent need for novel accurate prognostic biomarkers that could lead to more effective diagnostic and treatment strategies.

RNA modification could regulate genetic expression in a dynamic and reversible way. It is primarily modulated by three types of effector proteins: writers, readers, and erasers (Biswas and Rao, 2018). N6-Methylcytosine (m6A) is the main type of modification in eukaryotic cellular RNAs and plays a vital role in biological progress, including embryonic stem cell self-renewal, metabolism, immunity, and apoptosis (Meyer and Jaffrey, 2017). 5-Methylcytosine (m5C) is another common RNA modification. Similar to m6A methylation, m5C methylation is involved in RNA metabolism, structural stability, and stress response (Zhao et al., 2017). Furthermore, increasing evidence has shown that m5C modification can affect the progression of multiple malignant tumors, including HCC. Sun et al. reported that NSUN2-mediated m5C modification of long non-coding RNA (lncRNA) H19 was positively associated with poor differentiation of HCC (Sun et al., 2020). Cui et al. reported that NSUN4 was conspicuously upregulated in HCC and could work as an independent prognostic factor (Cui et al., 2022).

LncRNA is a type of non-coding RNA molecule with a length greater than 200 nt. It modulates gene expression mainly at epigenetic, transcriptional, and post-transcriptional levels (Bridges et al., 2021). Numerous lncRNAs have been reported to be closely correlated with carcinogenesis, metastasis, prognosis, and diagnosis of various cancers (Abbastabar et al., 2018). Previous studies have found that some methylation regulators could affect tumor progression by regulating the level of relevant lncRNAs. Dai et al. (2020) reported that METTL3 could upregulate the expression level of LINC00958 by increasing its stability, and LINC00958 sponged miR-3619-5p to upregulate hepatoma-derived growth factor, thereby promoting HCC progression. Hu et al. reported that IGF2BP2 could serve as a member of m6A readers and increase the stability of lncRNA DANCR, thus promoting cell proliferation and carcinogenesis of pancreatic cancer (Hu et al., 2020). In addition, Cui et al. reported that RNA m6A demethylase FTO could epigenetically upregulate the expression of LINC00022, thereby promoting tumorigenesis of esophageal squamous cell carcinoma (Cui et al., 2021). So far, few studies have reported the relationship between m5C regulators and lncRNAs in HCC progression and immune cell infiltration. Therefore, further understanding of how m5C modification interacts with lncRNAs in HCC may be favorable for exploring effective biomarkers and novel therapeutic targets.

Accumulating studies have shown that immune cells in the tumor microenvironment (TME) play a determinative role in tumor progression (Hinshaw and Shevde, 2019). A series of immunotherapy approaches have been successfully applied in clinical practices, such as the adoptive cell transfer, modulation of immune checkpoints, and dendritic cell-based vaccination (Lei et al., 2020). LncRNAs were key regulators in the immune system, which could regulate tumor invasion and evade immune surveillance by regulating tumor immune cell activation, proliferation, and cytokine secretion. In HCC, lncRNA FENDRR sponged miR-423-5p to suppress the inhibitory function of Tregs within TME, therefore weakening the immune evasion capability (Yu et al., 2019). Xue et al. (2019) reported that M2 macrophages were the predominant tumor-infiltrating immune cells in bladder cancer and associated with the prognosis of patients. However, the relationship between m5C-related lncRNAs and tumor-associated immune cells in HCC remains unknown.

This study aimed to explore the prognostic significance and immune landscape of the m5C-related lncRNAs in HCC. Based on the Cancer Genome Atlas (TCGA) database and bioinformatic analyses, we constructed an m5C-related lncRNA prognostic model and subsequently validated the accuracy and efficiency of the model. We utilized a nomogram to predict patients’ survival rates. Furthermore, the association between immune cell infiltration and the risk model was analyzed. More importantly, the responses of HCC patients to chemotherapy and immunotherapy were predicted to provide guidance for clinical treatment. Finally, we conducted experiments *in vitro* to identify the biological function of MKLN1-AS identified with the highest contribution in the risk model.

## Materials and methods

### Data and m5C regulator acquisition

The clinical and transcriptome data of 374 HCC tissues and 50 normal tissues were obtained from TCGA data website (http://portal.gdc.cancer.gov/). After excluding four samples without complete survival time and status, 370 HCC samples were included for further study. The clinical characteristics of these patients with HCC are shown in Supplementary Table S1. We also downloaded the annotation file of GRCH38 from the Ensemble official website (http://asia.ensembl.org) to distinguish mRNAs and lncRNAs. A total of 16 m5C regulators (NOP2, DNMT1, DNMT3A, DNMT3B, NSUN2, NSUN3, NSUN4, NSUN5, NSUN6, NSUN7, TRDMT1, ALYREF, YBX1, TET1, TET2, and TET3) were selected according to previous publications. The differential expression of 16 m5C regulators between tumor and normal tissues was analyzed using the limma package in R software (*p* < 0.05, | log2 (folding change) | > 1). We also used survival and survminer packages to perform survival analysis.

### Construction and validation of m5C-Related lncRNA prognostic risk model

Pearson correlation analysis was implemented to identify m5C-related lncRNAs with |Pearson R| > 0.4 and *p* < 0.001. We then used the limma package to perform differential m5C-related lncRNA expression analysis between HCC tissues and normal tissues and thus acquired 633 differentially expressed lncRNAs (*p* < 0.05). HCC cases were randomly divided into a training cohort and a testing cohort in a 1:1 ratio. In the training cohort, we conducted the univariate Cox regression analysis to screen out prognostic lncRNAs. Based on screened 17 lncRNAs with prognostic value, we performed the least absolute shrinkage and selection operator (LASSO) Cox regression and multivariate Cox regression to construct the prognostic prediction model. Five lncRNAs were extracted and used for further analysis. The risk score of each patient was calculated using the following formula:

Risk score = ∑_i=1_^n^Coef_i_ ×X_i_ (Coef_i_ represents the coefficients, and X_i_ represents the expression value of each m5C-related lncRNA).

Next, we graded each HCC patient. All patients were divided into high- and low-risk groups based on the median risk score calculated from the training cohort. We used the survival R package to implement Kaplan–Meier (KM) survival curve analysis. Receiver operating characteristic (ROC) curves was also constructed to evaluate the prognostic capability of the risk model. Moreover, we used principal component analysis (PCA) to visualize whether the risk score could well distinguish the high-risk group from the low-risk group.

### Evaluation of m5C-Related lncRNA risk model as independent prognostic indicator

We performed subgroup stratification survival analysis in clinicopathological features using KM plot to confirm the prediction performance of the model. Univariate and multivariate Cox regression analyses were conducted to assess whether the risk model was an independent factor. In addition, we constructed a heatmap based on clinical characteristics and differential expression of the five prognostic lncRNAs in different risk groups. Furthermore, combining the risk score and TNM stage, we established a nomogram to improve clinical diagnosis and application. Moreover, the nomogram’s predictive value was evaluated using ROC curve.

### Cell culture and quantitative real-time PCR assay

Human HCC cell lines (Huh7, HepG2, Hep3B, and SNU-387) and one normal liver cell line (L-02) were obtained from the Cell Bank of the Chinese Academy of Sciences (Shanghai, China). The cell lines were cultured in medium containing 10% fetal bovine serum (FBS) with 5% CO_2_ at 37°C. We also collected 20 pairs of HCC and para-carcinoma tissue samples from the Department of Hepatobiliary Surgery, the Affiliated Hospital of Xuzhou Medical University, from March 2021 to May 2022. To evaluate the expression level of m5C-related lncRNAs, we used RNA Isolater Total RNA Extraction Reagent (Vazyme, Nanjing, China) to isolate total RNAs from the tissue samples and cell lines. Reverse transcription was performed using HiScript II Q RT SuperMix (Vazyme, Nanjing, China), and quantitative real-time PCR was then conducted using ChamQ SYBR qPCR Master Mix (Vazyme, Nanjing, China). The relative expression of the five lncRNAs was calculated using the 2^−ΔΔCT^ method, and GAPDH served as an internal control. The primer sequences used in our study are listed in Supplementary Table S2.

### Prediction of m5C sites on five lncRNAs

RNAm5Cfinder (Ban et al., 2020), m5C-Atlas (Ma et al., 2022), and iRNA-m5C (Chen et al., 2021) databases were used to predict the m5C site of the lncRNAs.

### Function and signaling pathways enrichment analysis

The limma package was implemented to screen genes that were differentially expressed between the high- and low-risk groups. Subsequently, we performed gene ontology (GO) and Kyoto encyclopedia of genes and genomes (KEGG) analysis to explore the potential function and pathway between the differentially expressed genes (DEGs). Finally, GSEA software (GSEA_4.2.2) was used to identify potential signaling pathways in the high- and low-risk groups.

### Tumor immune analysis and somatic variant analysis

We calculated the correlation coefficient between the risk score and the immune infiltrated cells based on currently acknowledged software, including TIMER, XCELL, QUANTISEQ, MCPcounter, EPIC, CIBERSORT-ABS, and CIBERSORT. We used Wilcoxon signed-rank test to analyze the difference in immune infiltrating cell abundance between high- and low-risk groups. We also measured Spearman correlation coefficients between the risk score and the immune infiltrated cells, and the results are displayed herein in a lollipop diagram. The activities of 13 immune-related pathways between two groups were quantified using the “GSVA” package by ssGSEA. Next, we performed a two-way analysis of variance (ANOVA) to explore the association of the immune infiltration subtype with a risk score. R package maftools were used to analyze the gene somatic mutation data downloaded from the Genomic Data Commons (GDC) database.

### Immunotherapy response and drug sensitivity analysis

The TIDE algorithm was applied to predict the immunotherapeutic response. We also analyzed the differential expression level of 34 immune checkpoints between different risk groups. Furthermore, we used R package pRRophetic to predict the half-maximal inhibitory concentration (IC_50_) of drugs for HCC samples from different risk groups. In addition, the association between the expression level of prognostic lncRNAs and drug sensitivity was determined using relevant data obtained from CellMiner database.

### Cell transfection

SiRNAs targeting MKLN1-AS (si-MKLN1-AS#1, si-MKLN1-AS#2) and the negative control (si-NC) were designed and synthesized by Gene Pharma Technology (Shanghai, China). HepG2 cells were transfected with siRNAs by siLentFect Lipid Reagent (Bio-Rad, CA, United States). After 48 h, the cells were collected for further experiments. The siRNAs sequences against MKLN1-AS are listed in Supplementary Table S3.

### Cell counting Kit-8 (CCK-8) assay

Transfected cells (2000 cells/pore) were seeded into 96-well plates for CCK-8 assay. Then, 10 μl of CCK-8 reagent (APExBIO, USA) and 100 μl of serum-free MEM medium were introduced into cells and incubated for 2 h. Subsequently, the absorbance was measured at 450 nm at 0, 24, 48, 72, and 96 h.

### Transwell assay

In invasion assay, the top chamber was treated with Matrigel (BD Biosciences, Mississauga, Canada) while in the migration assay was not. Transfected cells (5 × 10^5^ cells/pore) were seeded into the upper layer of the transwell. A total of 700 μl chamber MEM medium with 20% FBS was added to the lower chamber, and the chamber was cultured at 37°C for 24–48 h. The invaded cells were fixed by 4% paraformaldehyde and stained with 0.1% crystal violet. A light microscope was used to observe cell migration and invasion.

### Wound healing assay

Transfected HepG2 cells were seeded in six-well plates and cultured to 80% confluence. Then, 200 μL pipette tips were used to create clear scratches in each well. Thereafter, the cells were cultured in a serum-free MEM medium. The scratches were imaged by a light microscopy at 0 and 24 h.

### Statistical analysis

One-way ANOVA was used to compare the differential expression level of 16 m5C regulators between HCC tissues and normal tissues. Cytoscape was used to plot the co-expression network of five m5C-related lncRNA–mRNA. The KM method and log-rank test were employed to compare the survival curves between various subgroups. Univariate and multivariate Cox regression analyses were used to identify independent prognostic factors. The nomogram was evaluated for predictable performance by calibration curve, and ROC curve was used to measure the prognostic efficiency of the nomogram for 1-, 3-, and 5-year overall survival (OS). Statistical analysis was carried out using R version 4.1.1, and *p* < 0.05 was considered statistically significant.

## Results

The Landscape of Expression and Prognosis of 16 m5C Regulators in HCC Tissues.

**FIGURE 1:**
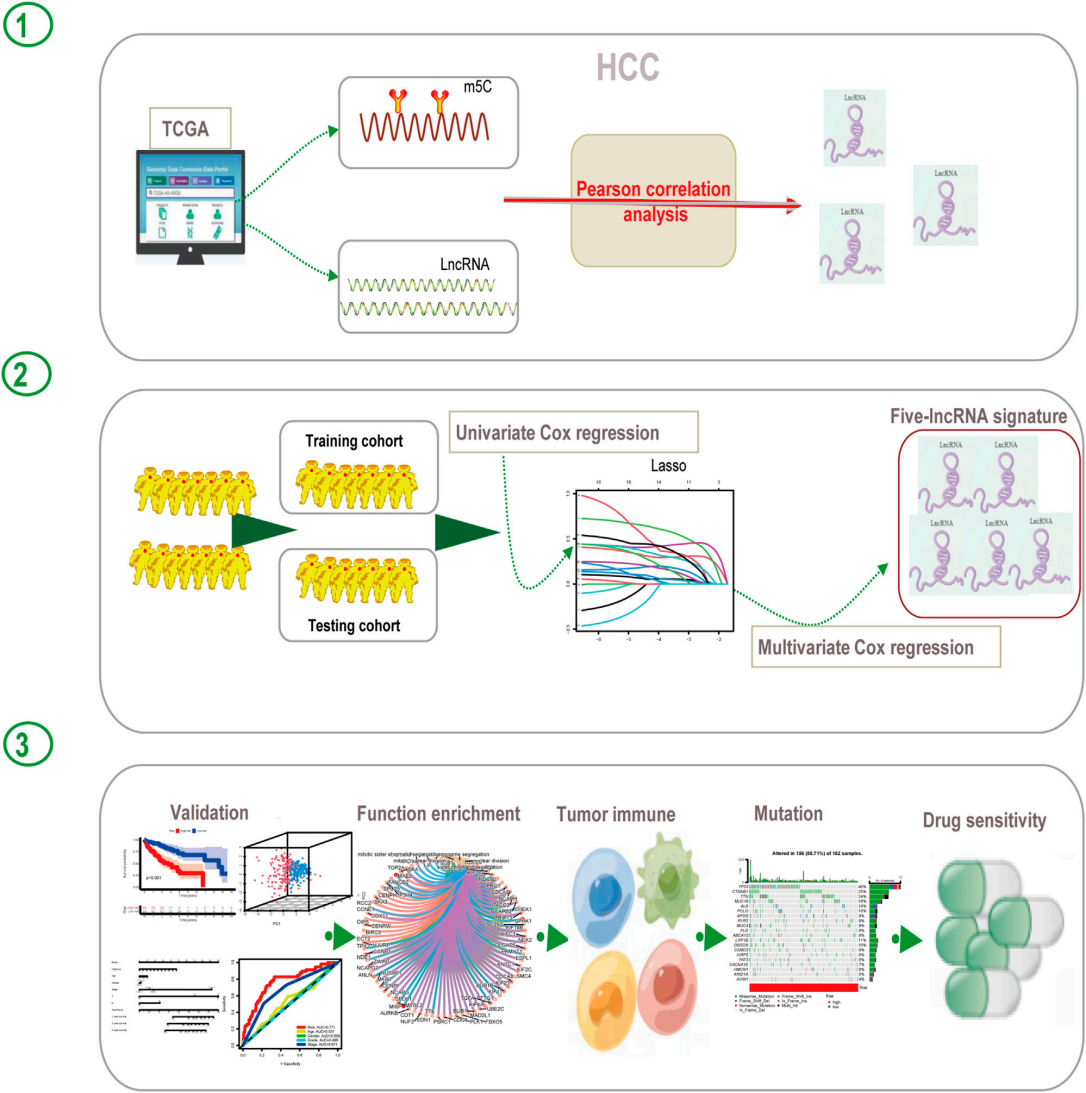
Flow diagram of this study.

**FIGURE 2:**
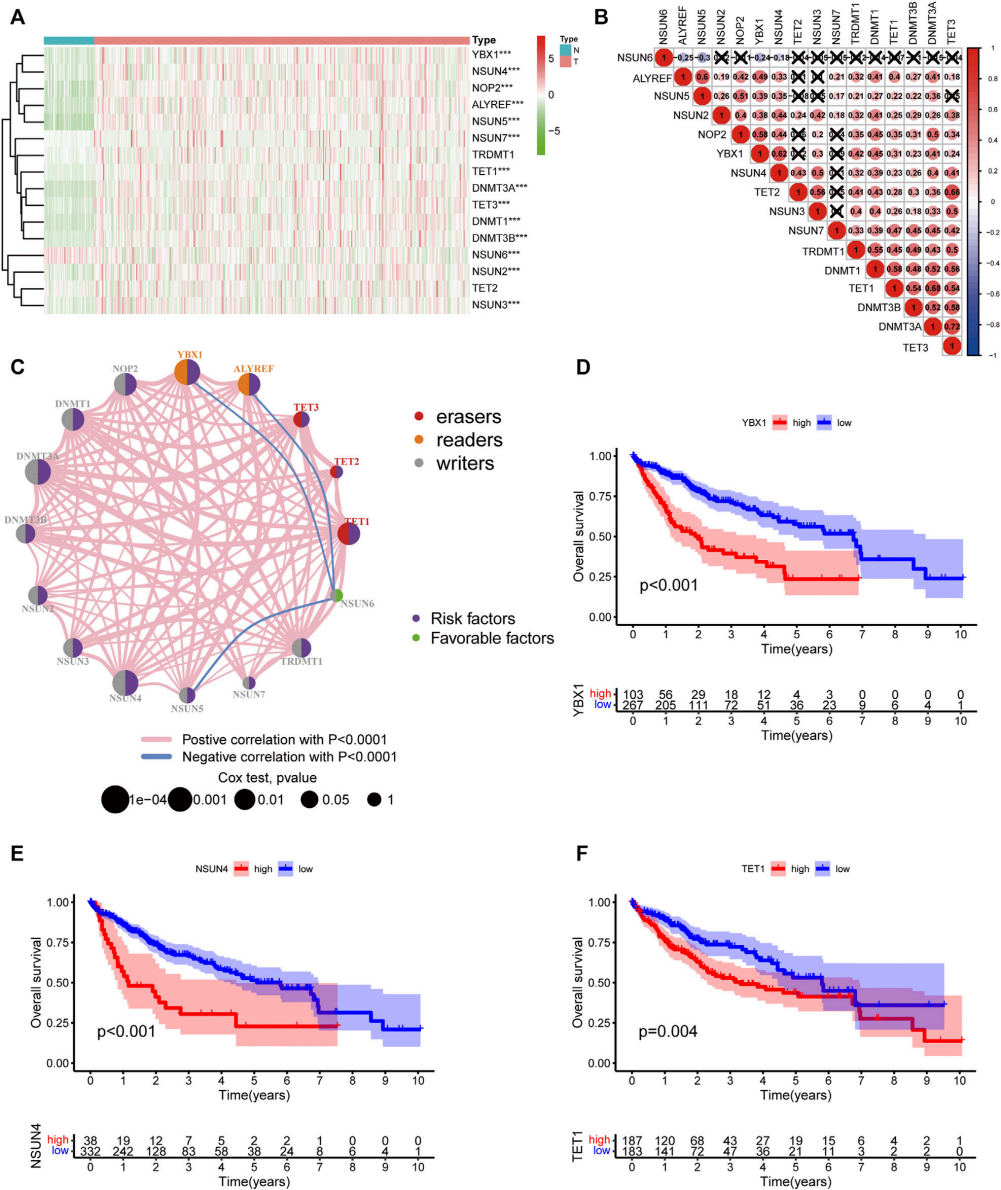
The landscape of expression and prognosis of m5C regulators in HCC patients. **(A)** Heatmap displaying different expressions of m5C regulators in HCC. **(B)** Spearman correlation analysis of 16 m5C regulators. **(C)** The interaction between m5C regulators in HCC. The size of the circle represented the influence of each regulator on prognosis, and the range of values calculated by log-rank test was *p* < 0.0001, *p* < 0.001, *p* < 0.01, *p* < 0.05, and *p* < 1. Purple in the right part of the circle indicates risk survival factors and green in the right part of the circle indicates favorable survival factors. The types of m5C regulators are labeled as different colors in the left part of the circle. The thickness of lines shows correlation strength. Positive correlation is shown in pink and negative correlation in blue. **(D–F)** Overall survival analysis based on three m5C regulators’ expression in HCC.

The workflow of this study is shown in Figure 1. We first explored the differential expression of 16 m5C regulators between HCC tissues and normal tissues in TCGA dataset. We found that all 16 m5C regulators except TET2 and TRDMT1 were differentially expressed. NSUN6 expression was significantly downregulated in HCC than in normal tissues, whereas that of the other 13 m5C regulators was significantly upregulated in HCC (Figure 2A). To evaluate the interaction among 16 m5C regulators, the correlation analysis showed that most m5C regulators were positively correlated with other regulators. We found a weak correlation between NSUN6 and other regulators and a strong correlation between DNMT3A and TET3 (Figure 2B). The m5C regulator network was depicted to indicate the interactions, connection, and prognostic value of m5C regulators for HCC patients. The most common positive correlation was found not only in the same category but also between different types of regulators. Negative correlations occurred between NSUN6 and NSUN5, NSUN6, and YBX1, and NSUN6 and ALYREF (Figure 2C). KM survival analysis showed significant differences among 15 m5C regulators in OS of HCC patients (Figures 2D–F and Supplementary Figure S1).

### Construction and verification of the m5C-Related lncRNA risk model

**FIGURE 3:**
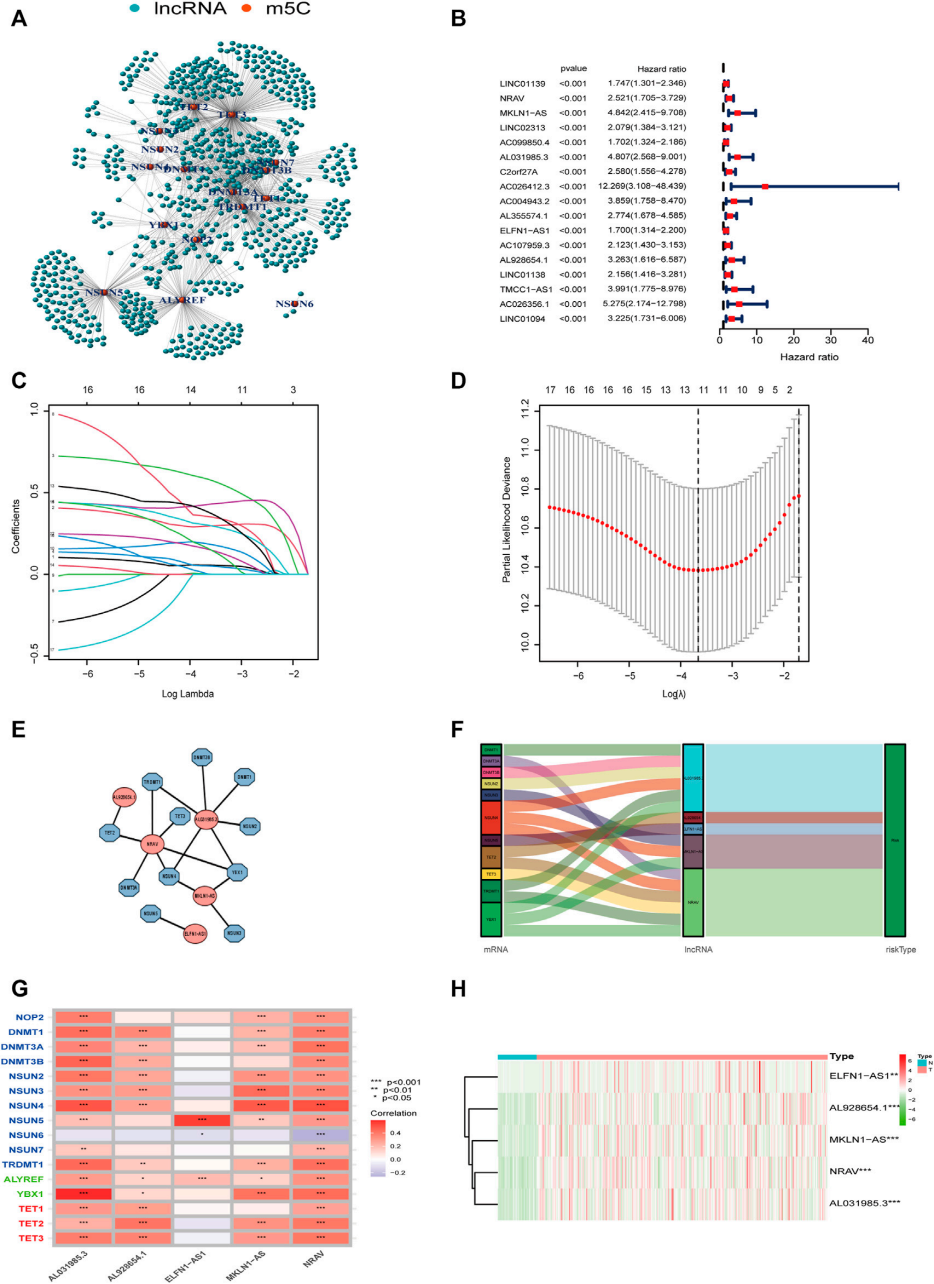
Construction of the m5C-lncRNA risk model. **(A)** The co-expression network of m5C regulators and their related lncRNAs. **(B)** Forest plot showing the hazard ratio of 17 lncRNAs with prognostic value using univariate Cox regression analysis. **(C,D)** LASSO regression is performed, and cross-validation for optimal parameter. **(E)** Co-expression network of the five m5C-related lncRNAs and m5C regulators. **(F)** Sankey diagram showing the relationship between m5C regulators and m5C-related lncRNAs. **(G)** The correlations between 16 m5C regulators and five m5C-related lncRNAs. **(H)** Heatmap of the differential expression of five lncRNAs in tumor-and normal tissues.

**FIGURE 4:**
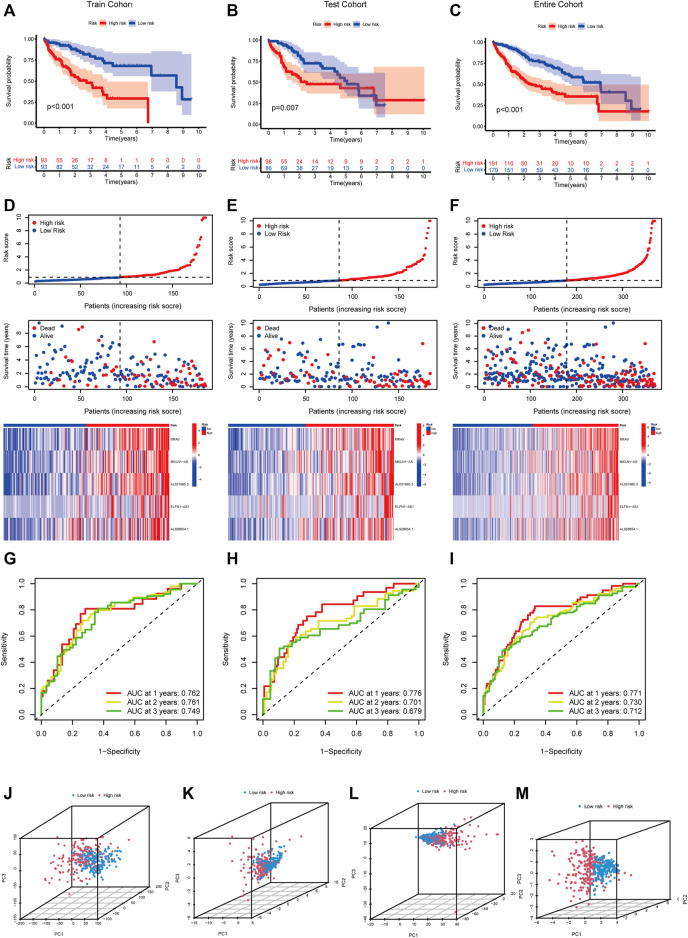
Verification of the m5C-lncRNA risk model. Kaplan-Meier curves of overall survival of high-risk and low-risk groups in the training cohort **(A)**, testing cohort **(B)**, and entire cohort **(C)**. The distribution of risk scores, survival status and expression matrix of five-lncRNA signature in the training cohort **(D)**, testing cohort **(E)**, and entire cohort **(F)**. ROC curves of the model for OS prediction including 1, 2, and 3 years in the training cohort **(G)**, testing cohort **(H)**, and entire cohort **(I)**. PCA analysis between the high-risk and low-risk groups based on all genes **(J)**, m5C genes **(K)**, m5C-lncRNAs **(L)**, and risk lncRNAs **(M)**.

Pearson correlation analysis was conducted to identify the m5C-related lncRNAs based on the expression of m5C regulators and lncRNAs in HCC patients. Then 633 m5C-related lncRNAs were screened out using differential expression analysis. We constructed a co-expression network of m5C regulators and their related lncRNAs (Figure 3A). In addition, we randomly divided 370 HCC cases into a training cohort (50%, *n* = 186 cases) and a testing cohort (50%, *n* = 184 cases). Next, univariate Cox regression analysis was conducted to screen the prognostic m5C-related lncRNAs in the training cohort. The result showed that 17 lncRNAs with increased risk (hazard ration, HR > 1) were deemed to have important prognostic value (Figure 3B). Subsequently, we performed LASSO Cox regression to analyze the 17 prognostic m5C-related lncRNAs, followed by multivariate Cox regression analysis to build a prognostic risk model for HCC (Figures 3C,D). Finally, we obtained five lncRNAs with a prognostic significance to construct the prognostic model (Supplementary Table S4). A co-expression network for the visualization of the five m5C-related lncRNAs and 16 m5C regulators was established (Figures 3E,F). We also observed that NRAV and AL031985.3 had the strongest correlation with m5C regulators, whereas ELFN1-AS1 had the weakest correlation. Moreover, correlations among m5C regulators and lncRNAs were mostly positive (Figure 3G). As displayed in Figure 3H, the expression levels of the five m5C-related lncRNAs were significantly different between HCC and normal tissues. The risk score of each HCC patient was calculated as follows: Risk score = 0.4635* NRAV expression level +0.8199* MKLN1-AS expression level +0.6452* AL031985.3 expression level + 0.3553* ELFN1-AS1 expression level +0.7350* AL928654.1 expression level. Notably, the positive coefficients of the five lncRNAs revealed that they were all risk survival factors. We then divided the patients of the training cohort into high- and low-risk groups based on the median risk score. KM survival curves showed that patients with high-risk scores had poor prognoses (Figure 4A). Risk score and survival status distributions showed that more and more patients died as the risk score increased. Additionally, our analysis showed that all the five lncRNAs had higher expression levels in the high-risk group (Figure 4D). Then, we used the same score formula to calculate the risk score of each patient in the testing cohort and the entire cohort, which were employed to validate the signature. The results were similar to those displayed in the training cohort (Figures 4B,C,E,F). Furthermore, we analyzed the prognostic accuracy of risk score using the ROC analysis (in the training cohort: 1-, 2-, and 3-year AUC = 0.762, 0.761, and 0.749, respectively; in the testing cohort: 1-, 2-, and 3-year AUC = 0.776, 0.701, and 0.679, respectively; in the entire cohort: 1-, 2-, and 3-year AUC = 0.771, 0.730, and 0.712, respectively) (Figures 4G–I). We used PCA to visualize the different distribution patterns between the two groups based on all genes, m5C genes, m5C-lncRNAs, and risk lncRNAs. Based on risk lncRNAs, patients were distributed in obviously different directions, so that the m5C-related lncRNA risk model may well differentiate between the high- and low-risk groups (Figures 4J–M).

### Validation of the suitability of the model using stratified survival analysis

We conducted stratified analysis by dividing the HCC patients into various subgroups and comparing the OS between high- and low-risk groups to evaluate the prognostic value of this model under different HCC clinicopathological subgroups. The survival analysis revealed that patients with high-risk scores had shorter OS in various subgroups (age >65 years *versus* age ≤65 years, female *versus* male, G1–2 *versus* G3–4, T stage1–2 *versus* T stage3–4, M0 stage, N0 stage, TNM stage I–II *versus* TNM stage III–IV) (Supplementary Figure S2).

### The m5C-Related lncRNA risk model was an independent prognostic factor for HCC patients

**FIGURE 5:**
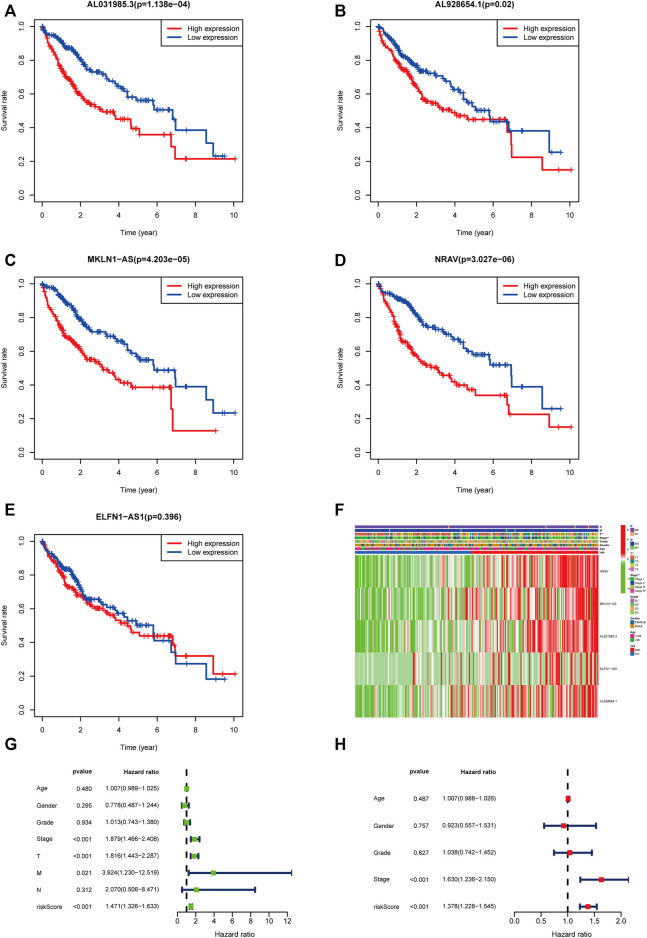
Validation of the m5C-related lncRNAs risk score as an independent prognostic factor in HCC patients. **(A–E)** KM survival curves indicated the relationship of the five lncRNAs with prognosis in HCC patients. **(F)** Heatmap showing the correlation between expression levels of the five m5C-lncRNAs and clinicopathological features. **(G,H)** Univariate and multivariate Cox regression analysis of risk score and clinicopathological parameters.

**FIGURE 6:**
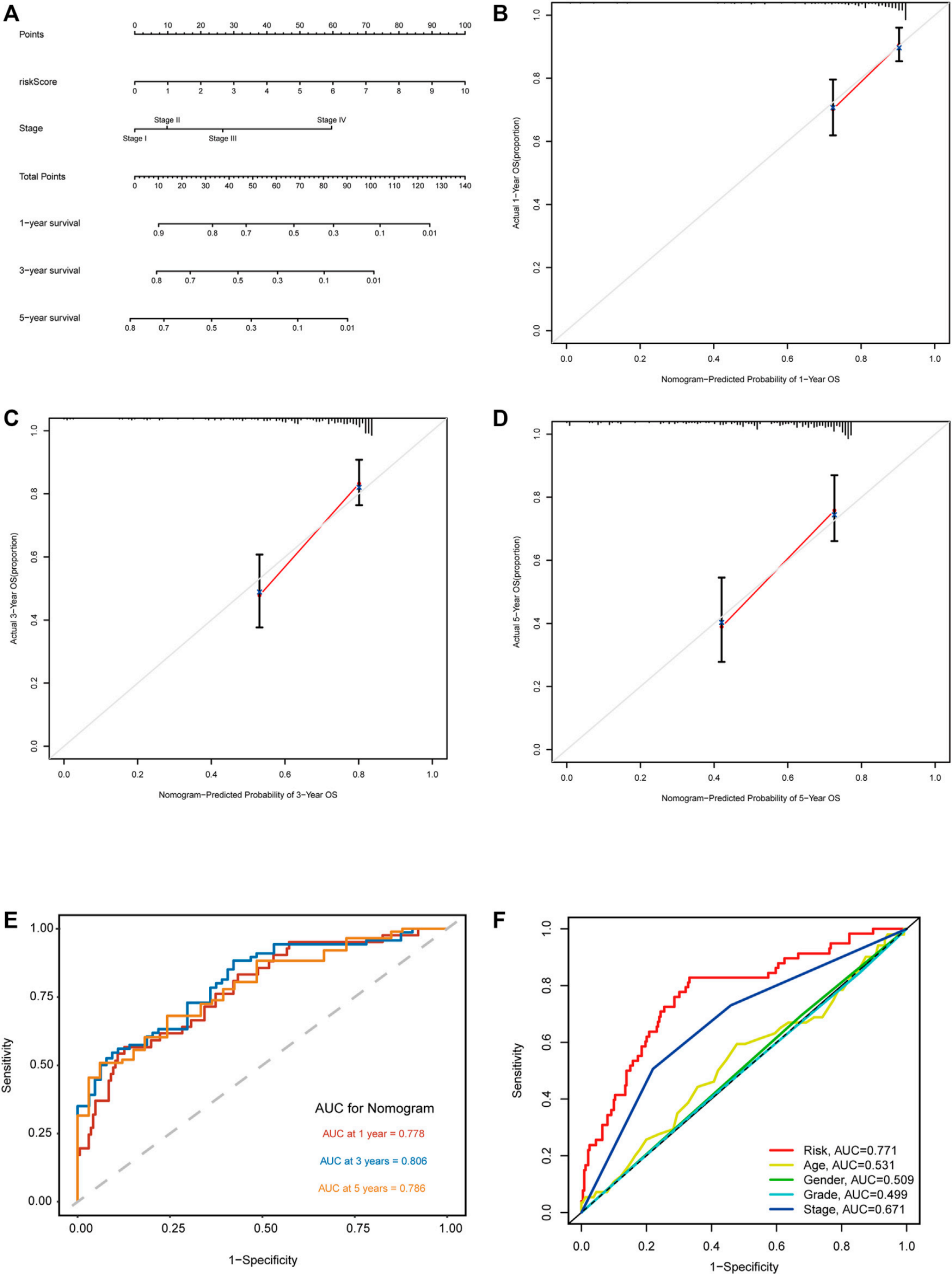
Construction and validation of the nomogram based on m5C-related lncRNA risk model. **(A)** Nomogram with risk score and TNM stage for predicting 1-, 3-, and 5-year survival for HCC patients. **(B–D)** The calibration curves showing the consistency of nomogram-predicted and actual 1-, 3-, and 5-year OS. **(E)** ROC analysis evaluating the predictability of the nomogram for 1, 3, and 5 years OS. **(F)** A comparison of AUC of risk score and clinical factors at 1-year showed the optimal prognostic value of the risk score.

According to the expression level of each lncRNA, we divided HCC patients into high- and low-expression groups and then performed KM survival analysis on them. The survival curves showed that patients in the high-expression group of AL031985.3, AL928654.1, MKLN1-AS, and NRAV had shorter OS and worse prognoses. Nevertheless, OS of ELFN1-AS1 in the high- and low-expression groups had no statistical differences (Figures 5A–E). According to the heatmap, TNM and T stages (*p* < 0.01) were statistically significantly different between the high- and low-risk groups, but other clinicopathological features had no statistical differences (Figure 5F). Furthermore, we conducted univariate and multivariate Cox regression analyses to confirm whether the risk score calculated using the m5C-related lncRNA risk model could be used as an independent prognostic factor. The univariate analysis showed that TNM stage (*p* < 0.001), T stage (*p* < 0.001), M stage (*p* = 0.021), and risk score (*p* < 0.001) were prognostic factors, whereas the multivariate Cox regression analysis revealed that TNM stage (*p* < 0.001) and risk score (*p* < 0.001) could serve as independent prognostic factors for patients with HCC (Figures 5G,H). In clinical practices, to provide an accurate quantitative tool for evaluating the individual OS of HCC patients, we formulated a nomogram based on risk score and TNM stage screened by multivariate Cox regression analysis to predict 1-, 3-, and 5-year OS probability (Figure 6A). As shown in the calibration curve, the actual and predicted 1-, 3-, and 5-year OS were almost in perfect concordance (Figures 6B–D). The time-dependent ROC curves were used to evaluate the specificity and sensitivity of the nomogram for predicting the prognosis of HCC patients. Our results revealed that AUC values of nomogram were 0.778, 0.806, and 0.786 at 1-, 3-, and 5-year OS, respectively (Figure 6E). Besides, we compared AUC values of risk score, age, gender, grade, and stage and noted that the risk score was superior to other clinical factors (Figure 6F). In summary, the m5C-related lncRNA risk model had the optimal ability to predict the prognosis of HCC patients.

### Validation of the five m5C-Related lncRNA expression in hepatocellular carcinoma cell lines and tissues, and analysis of m5C modification sites

**FIGURE 7:**
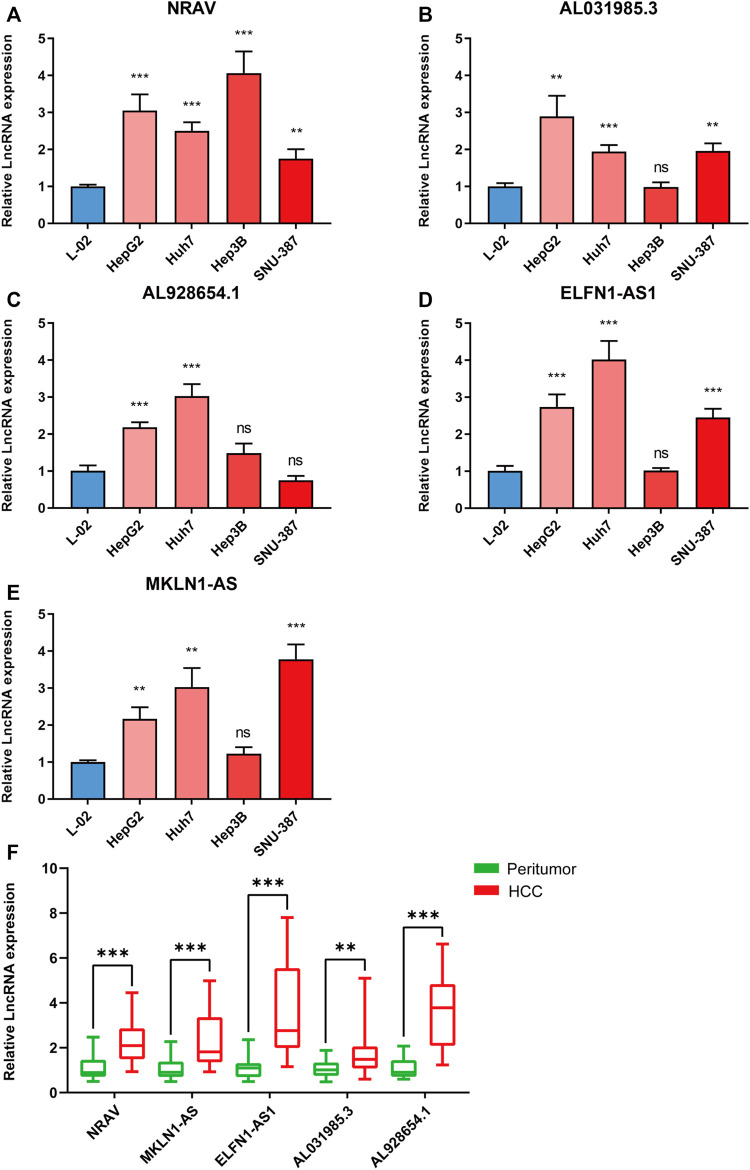
Validating the expression levels of five m5C-related lncRNAs. The expression levels of m5C-related lncRNAs in **(A–E)** 5 cell lines and **(F)** 20 pairs HCC tissues and paracancerous tissues. *p < 0.05, **p < 0.01, and ***p < 0.001.

We further validated the five m5C-related lncRNA expression levels in HCC cell lines and tissue samples by RT-qPCR assay. The expression levels of these five lncRNAs were examined in Huh7, HepG2, Hep3B, SNU-387, and L-02 cell lines. Our results showed that NRAV expression level was upregulated in HCC cell lines compared with the liver cell line (Figure 7A). AL031985.3, AL928654.1, ELFN1-AS1, and MKLN1-AS expressions were upregulated in part of HCC cell lines (Figures 7B–E). We then performed the differential expression analysis of the five lncRNAs in 20 pairs of HCC and para-carcinoma tissue samples. The results revealed that MKLN1-AS, NRAV, ELFN1-AS1, AL928654.1, and AL031985.3 expression levels were upregulated in HCC tissues (Figure 7F). After scanning the m5C-Atlas, we found two m5C modification sites on NRAV and eleven m5C modification sites on MKLN1-AS. We also utilized RNAm5Cfinder and iRNA-m5C databases to predict potential m5C modification sites on our five lncRNAs, and eventually obtained m5C modification sites on all five lncRNAs (Supplementary Table S5).

### The functional and pathway enrichment analysis

**FIGURE 8:**
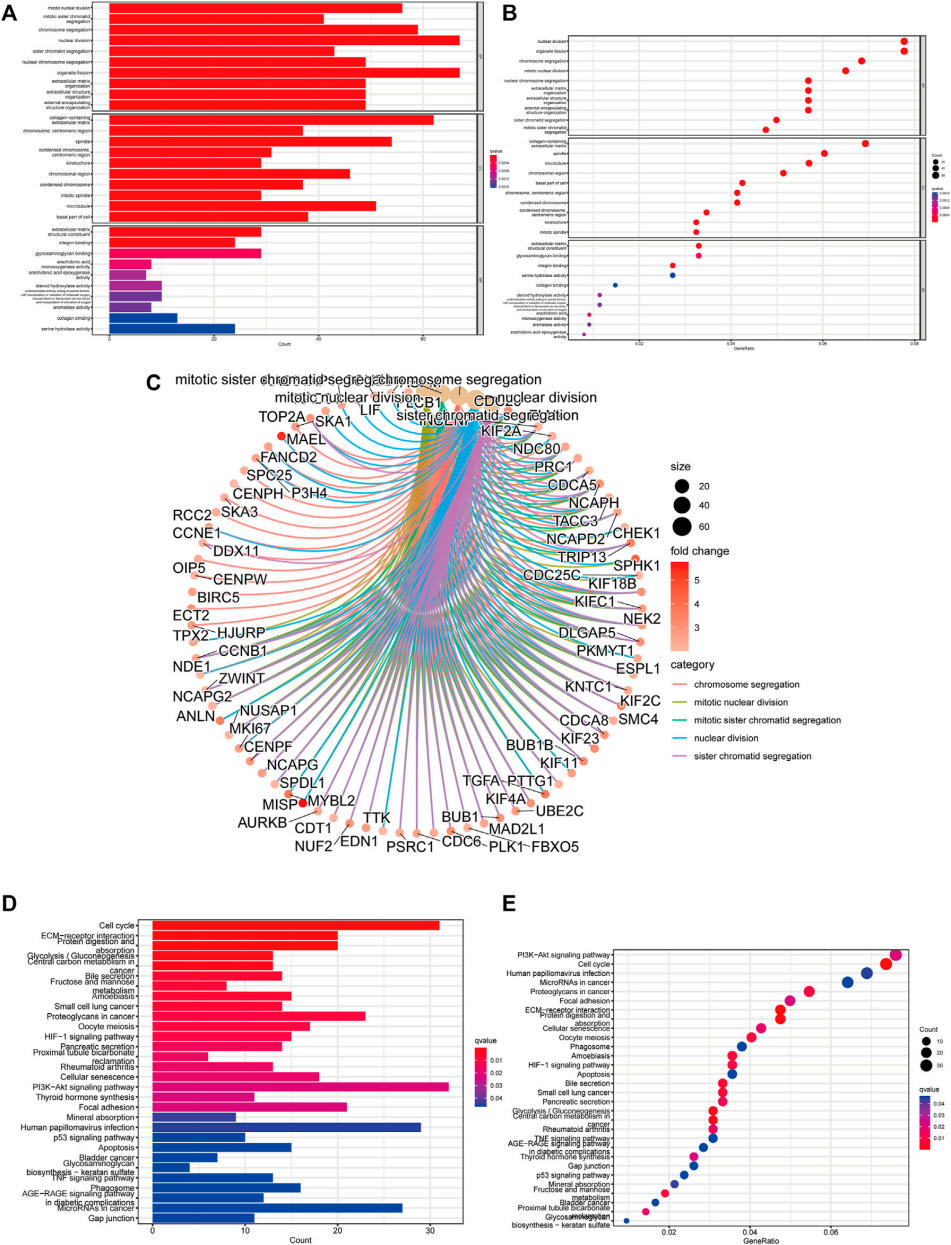
Function and pathways enrichment analysis of m5C-related lncRNAs. **(A–C)** Visualization of the enriched biological processes by GO analysis. **(D,E)** KEGG analysis displaying the enriched signaling pathways related to risk model.

We conducted GO and KEGG analysis based on the differential genes between the high- and low-risk groups to better identify the potential biological mechanisms. The top five GO terms were sister chromatid segregation, nuclear division, mitotic sister chromatid segregation, mitotic nuclear division, and chromosome segregation (Figures 8A–C). KEGG analysis showed that these signaling pathways were mainly enriched in cell cycle, PI3K-Akt signaling pathway, proteoglycans in cancer, glycolysis/gluconeogenesis, and ECM–receptor interaction (Figures 8D,E). Furthermore, the activated pathways enriched in the high- and low-risk groups were identified through gene set enrichment analysis (GSEA). We found that Notch signaling pathway, cell cycle, regulation of autophagy, and pathways in cancer were activated in the high-risk group, whereas fatty acid metabolism, tryptophan metabolism, PPAR signaling pathway, and beta alanine metabolism were activated in the low-risk group (Supplementary Figure S3). These results revealed the association of m5C-related lncRNAs with biological function in HCC.

### Association of m5C-Related lncRNAs with immune cell infiltration

**FIGURE 9:**
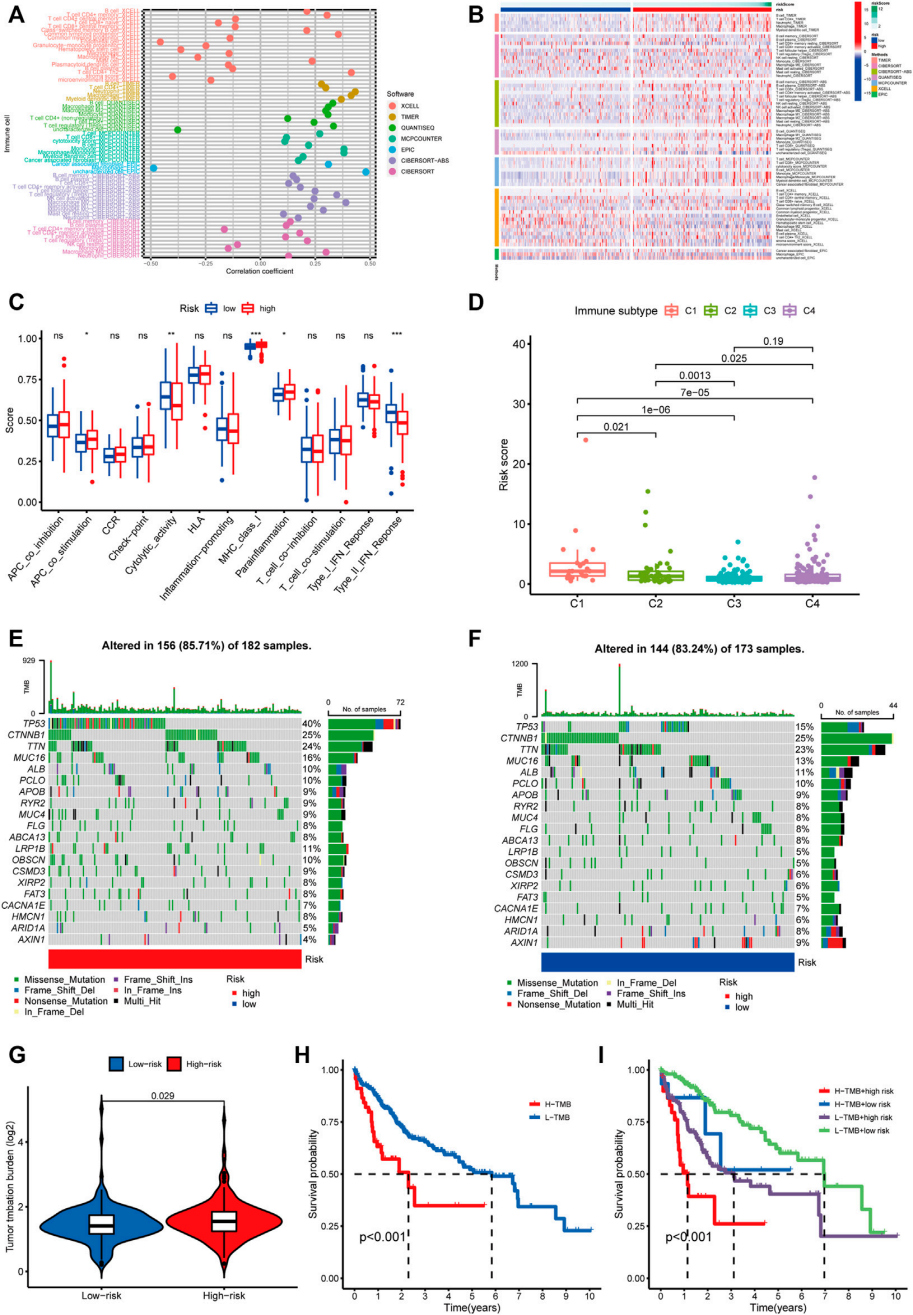
Estimating the correlation between m5C-related lncRNAs and immune infiltration and mutation analysis. **(A)** The correlation analysis of risk score and tumor-infiltrating immune cells by TIMER, XCELL, QUANTISEQ, MCPcounter, EPIC, CIBERSORT-ABS, and CIBERSORT software. **(B)** A heatmap indicating the differential immune responses between the high- and low-risk groups based on the above seven software. **(C)** The differential scores of 13 immune-related functions in high- and low-risk groups. **(D)** Comparison of the risk score in different immune infiltration subtypes. *p < 0.05, **p < 0.01, and ***p < 0.001; ns, non-significant. **(E,F)** Waterfall plot of the 20 top mutated genes with high mutation frequency in the high-risk group **(E)** and low-risk group. **(F,G)** The different mutation event between two groups. **(H)** KM analysis between high/low TMB groups. **(I)** Comparative analysis of prognosis combining risk score and TMB.

We conducted a Spearman correlation analysis to illustrate the relationship between the m5C-related lncRNAs and immune cell infiltration. As shown in the lollipop diagram, the risk score was positively correlated with Treg cells, CD4 + T cells, neutrophils, M1 macrophages, and M2 macrophages and negatively correlated with hematopoietic stem cells and endothelial cells (Figure 9A and Supplementary Table S6). The heatmap indicated the difference in the infiltrating levels of immune cells between the high- and low-risk groups based on the TIMER, XCELL, QUANTISEQ, MCPcounter, EPIC, CIBERSORT-ABS, and CIBERSORT software (Figure 9B). Comparative analysis of immune-related functions or pathways by ssGSEA showed that the scores of APC co-stimulation, MHC class I and para-inflammation were higher in the high-risk group, while the cytolytic activity and type II IFN response scores were the opposite (Figure 9C). Furthermore, we compared the risk score in different immune infiltration subtypes and found that the high-risk score was strikingly correlated with C1, while the low-risk score was strikingly correlated with C4 (Figure 9D). The above results suggested that the m5C-related lncRNA risk model of HCC was related to immune status.

### Tumor mutation burden based on m5C-Related lncRNA risk model

We analyzed the association between the risk score and tumor mutation burden (TMB) using somatic mutation information downloaded from TCGA-HCC cohort. Figures 9E,F show the top 20 mutated genes with a high mutation frequency. We found that patients in the high-risk group had more mutation event compared with those in the low-risk group (Figure 9G), and TP53 presented the highest mutation frequency in both groups. Besides, patients with high TMB suffered shorter survival time than those with low TMB (Figure 9H). Next, we divided HCC patients into four groups to conduct a combined analysis of TMB and risk score: high TMB + high risk, high TMB + low risk, low TMB + high risk, and low TMB + low risk. As shown in Figure 9I, patients in the low TMB + low-risk group were found with a better survival probability than those in the other three groups.

### Evaluation of responses to immunotherapy and chemotherapy based on m5C-Related lncRNA risk model

**FIGURE 10:**
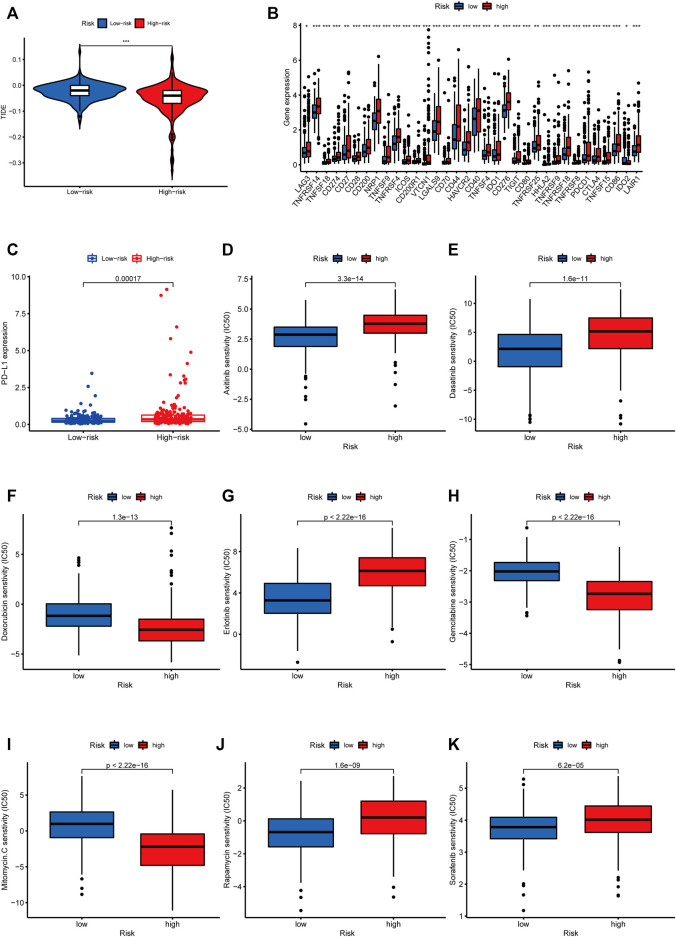
Analysis of immunotherapy and chemotherapy responses based on m5C-related lncRNAs risk model. **(A)** Comparison of TIDE scores between the high-risk and low-risk groups. **(B)** The difference of 34 immune checkpoints expression level between high- and low-risk groups shown in the box plot. **(C)** Differences in PD-L1 expression between high- and low-risk groups. IC_50_ of axitinib **(D)**, dasatinib **(E)**, doxorubicin **(F)**, erlotinib **(G)**, gemcitabine **(H)**, mitomycin.C **(I)**, rapamycin **(J)**, and sorafenib **(K)** in high- and low-risk groups.

**FIGURE 11:**
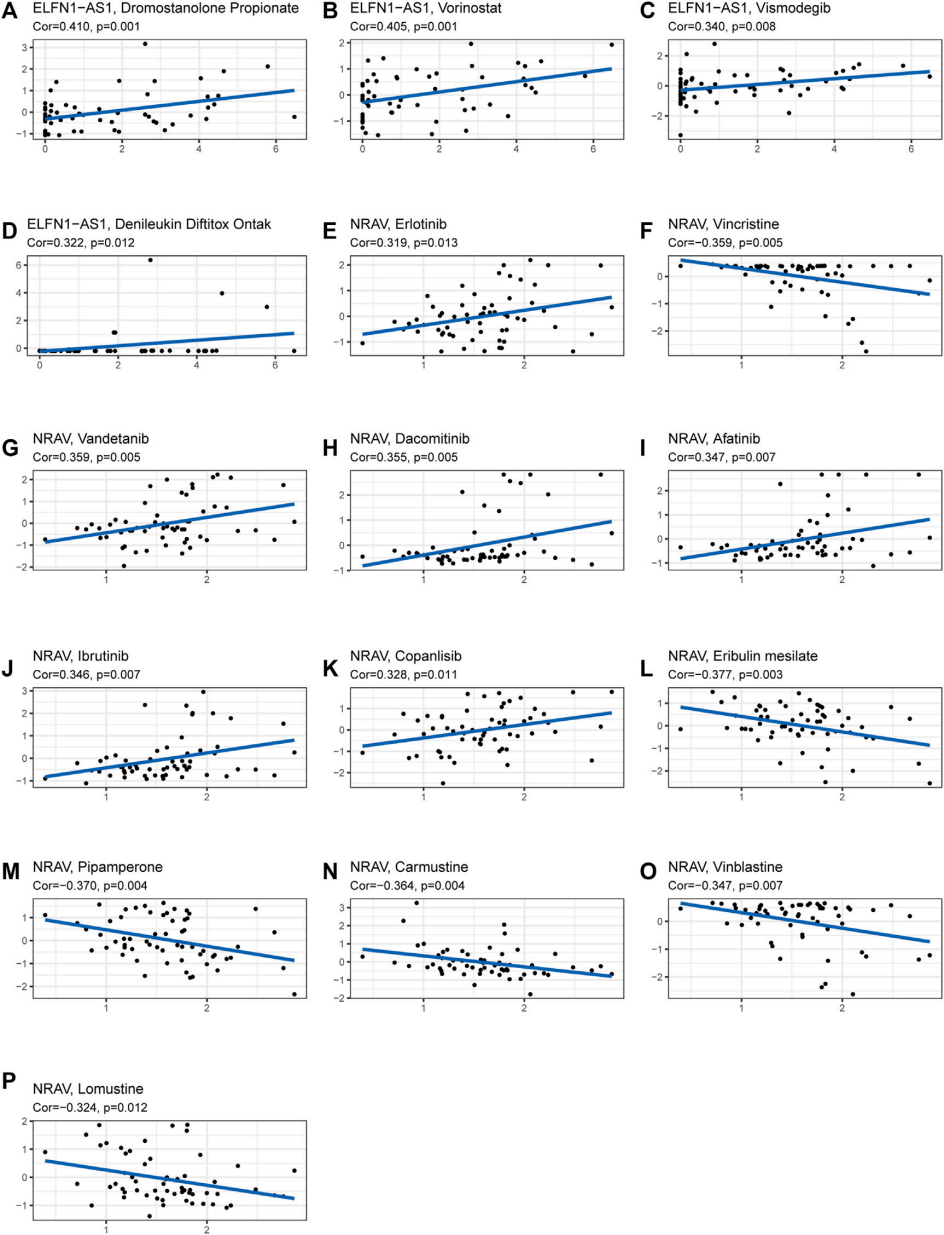
Analysis of correlation between the prognostic lncRNAs expression and drug sensitivity. **(A–P)** The scatter plot showed the top 16 associations between prognostic lncRNAs expression and drug sensitivity.

The TIDE algorithm was used to predict immunotherapy response in the high- and low-risk groups. As demonstrated in Figure 10A, the patients in the high-risk group were found with higher TIDE scores than those in the low-risk group, suggesting that the high-risk group was more likely to react to immunotherapy. To investigate the relationship between the risk group and the expression of immune checkpoints, we compared the expression levels of 34 immune checkpoints and found higher expression level in the high-risk group than in the low-risk group (Figure 10B). Recently, immune checkpoint inhibitors (ICIs) have been conducted in the field of HCC therapy. Programmed cell death 1 ligand 1 (PD-L1), one of the key indicators in cancer immune evasion, has already been used to predict the potential response to immune checkpoint blockade (ICB) therapy. In our study, we discovered that PD-L1 expression level was significantly higher in the high-risk group than in the low-risk group, indicating that high-risk patients were more sensitive to PD-L1 blockade immunotherapy (Figure 10C). Furthermore, we identified the relationship between risk score and common chemotherapeutic drug sensitivity. The results showed that IC_50_ values of axitinib, rapamycin, dasatinib, sorafenib, and erlotinib were higher in the high-risk group, suggesting that patients from the low-risk group had higher sensitivity to these five drugs. Besides, IC_50_ values of gemcitabine, doxorubicin, and mitomycin C was higher in the low-risk group, which indicated higher sensitivity to these three drugs in the high-risk group (Figures 10D–K). Finally, we investigated the prognostic lncRNAs from the CellMiner database NCI 60 RNA seq and compound activity: DTP NCI-60. As revealed in Figure 11, ELFN1-AS1 and NRAV were correlative to the sensitivity of some chemotherapy drugs, and the correlativity between ELFN1-AS1 expression level and the sensitivity of drug dromostanolone propionate was the strongest (correlation = 0.410, *p* = 0.001).

### Functional validation analysis

**FIGURE 12:**
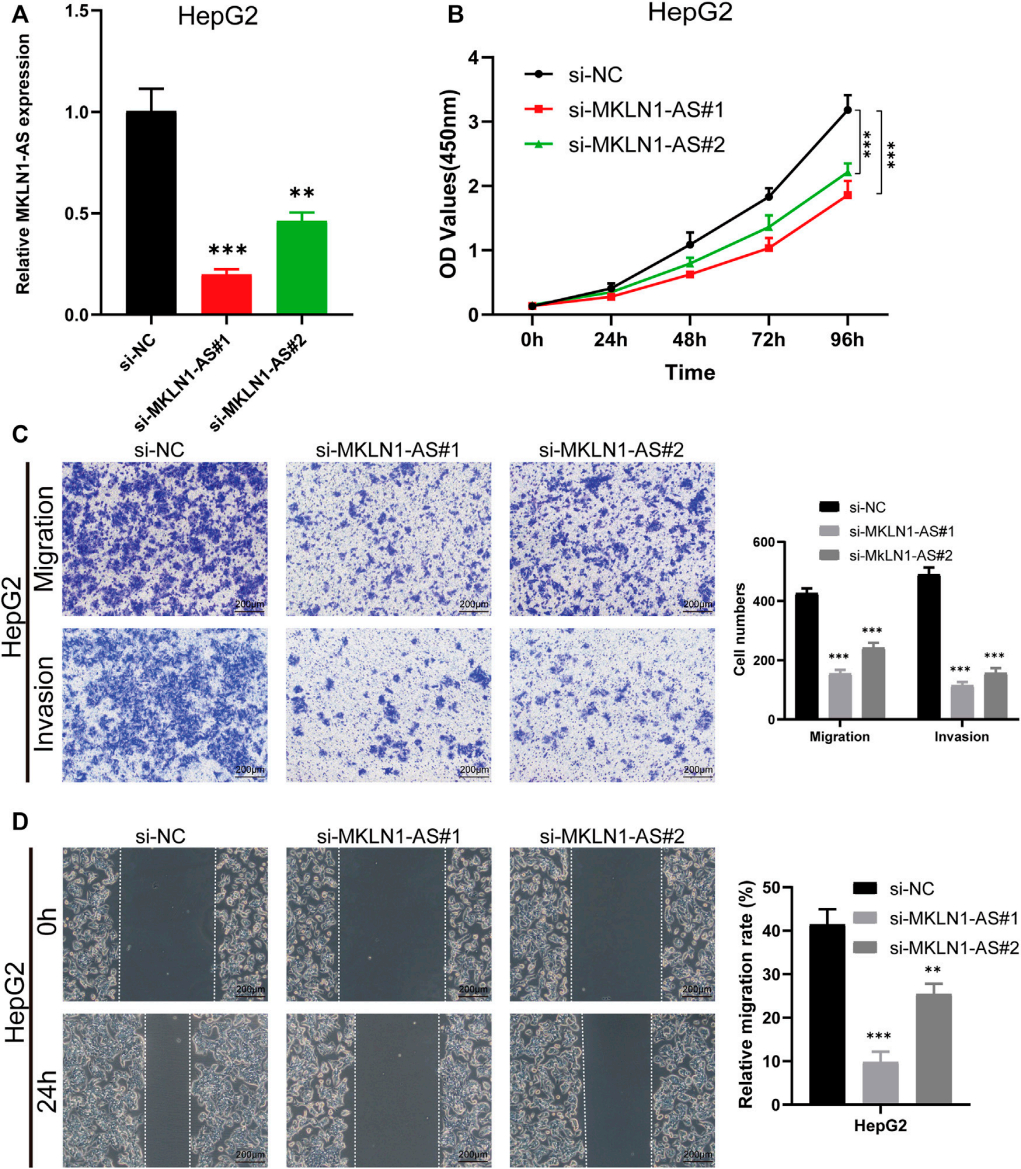
MKLN1-AS facilitated the proliferation, migration, and invasion of HCC cells *in vitro*. **(A)** qRT-PCR validation of MKLN1-AS expression in HepG2 cells transfected with siRNAs. **(B)** The viability of HepG2 detected by the CCK-8 assay. **(C)** Transwell assay performed to evaluate the migration and invasion abilities of HepG2 cell transfected with indicated siRNAs. **(D)** Cell migration ability detected *via* wound healing assay. All data are presented as the mean ± standard deviation (SD). * *p* < 0.05, **p < 0.01, ***p < 0.001.

We then selected MKLN1-AS with the highest contribution in the risk model (Coef = 0.8) for further biological function verification in HCC cells. HepG2 cell was chosen for MKLN1-AS knockdown *via* transfection with siRNAs. qRT-PCR assays were performed to detect the transfection efficiency, and both siRNA fragments downregulated the expression level of MKLN1-AS (Figure 12A). CCK-8 assay indicated that MKLN1-AS knockdown markedly repressed the proliferation of HepG2 cells (Figure 12B). Then, we observed that the knockdown of MKLN1-AS remarkably suppressed migration and invasion abilities of HepG2 cells *via* transwell assay (Figure 12C). Furthermore, wound healing assay showed that after culture for 24 h, scratches of knock-down groups healed slowly and the area of cell migration decreased, indicating that downregulation of MKLN1-AS expression could inhibite the migratory ability of HepG2 cells (Figure 12D). Collectively, these findings confirmed that MKLN1-AS promotes HCC cell proliferation, migration, and invasion *in vitro*.

## Discussion

RNA post-transcriptional modifications (such as m6A, m5C, m1A, and m7G), as well-explored events, have been proved to be involved in the carcinogenesis and progression of various cancers. M5C modification, already observed in various RNAs, can promote the proliferation, migration, invasion, and angiogenesis of cancers (Li et al., 2022). LncRNAs, which are widely used as a target or biomarker for disease and treatment, can regulate tumor growth through various mechanisms, including chromatin remodeling, natural antisense transcripts, and chromatin interactions (Fang and Fullwood, 2016). A growing body of evidence has indicated that m6A modification can modulate lncRNAs to affect the pathological processes of cancer development. However, few studies have systematically reported the function of m5C-related lncRNAs in HCC. Taken together, gaining more insight into the relationship between lncRNAs and m5C has a meaningful likelihood of predicting the prognosis and guiding therapy for HCC. In this study, we constructed a prognostic risk model of five m5C-related lncRNAs and analyzed their role in the prognosis and immune cell infiltration. Moreover, cell experiments for one of the five m5C-related lncRNAs, MKLN1-AS, were conducted to confirm the accuracy of the prognostic risk model. So far, no study has been conducted to analyze the prognostic value of m5C-related lncRNAs in HCC. Our findings may be used as novel biomarkers or therapeutic targets for more accurate diagnosis, prognosis, and treatment.

Recently, ferroptosis-related gene signature, pyroptosis-related lncRNA signature, inflammatory response-related gene signature, immune-related gene signature, and m6A-related gene signature have been constructed to predict OS for HCC. In this study, we explored m5C-related lncRNAs by analyzing HCC data downloaded from TCGA database, and five m5C-related lncRNAs capable of prognostic value were finally selected to construct a prognostic risk model. PCA analysis showed that high-risk group patients could be clearly differentiated from the low-risk group patients by using the model. Besides, the model can serve as an independent prognostic factor for HCC patients based on univariate and multivariate Cox regression analyses. In addition, our nomogram could figuratively predict 1-, 3-, and 5-year survival according to the comprehensive score. The results above suggested that the prognostic risk model constructed by five lncRNAs had a potential predictive effect. The five m5C-related lncRNAs, which were NRAV, AL031985.3, MKLN1-AS, ELFN1-AS1, and AL928654.1, were highly expressed in tumor tissues by bioinformatics analysis. We subsequently validated the expressions of the five lncRNAs in HCC cell lines and tissues by RT-qPCR assay. The results were consistent with results from bioinformatics analysis. Besides, four of these lncRNAs were associated with prognosis based on survival analysis. A recent study has revealed that NRAV could negatively regulate antiviral responses by repressing the expression of interferon-stimulated genes (Ouyang et al., 2014). MKLN1-AS has been proven to be one of lncRNAs in hepatocellular carcinoma-related competing endogenous RNA networks and affected HCC progression (Gao et al., 2020). Our results showed that the knockdown of MKLN1-AS could suppress proliferation, migration, and invasion in the HepG2 cell line. Bioinformatic analysis showed that AL031895.3, as inflammatory response-related lncRNA and immune-related gene, was also overexpressed in HCC cell lines, which indicated that AL031985.3 could be an adverse prognostic indicator for HCC (Li et al., 2022). ELFN1-AS1 affects the proliferation, invasion, and metastasis of esophageal cancer and colorectal cancer by regulating miRNAs (Zhang et al., 2020; Zhai et al., 2021). AL928654.1 has not been reported yet; hence, further studies are needed to clarify the effects of these five lncRNAs in the tumorigenesis and development of HCC.

Using GSEA, we explored the molecular mechanism underlying m5C-related lncRNAs. Notch signaling pathway, cell cycle, regulation of autophagy, and pathways in cancer were significantly enriched in the high-risk group. Previous studies have shown that Notch signaling pathway was related to the pathogenesis of liver fibrosis, and EGFL8 regulated HCC cell migration, invasion, and apoptosis *via* the activation of Notch signaling pathway (Wu et al., 2021; Zhu et al., 2021). The cell cycle regulates the duplication and transmission of genetic information; however, the dysregulated cell cycle progression is common in the pathogenesis of cancer (Wiman and Zhivotovsky, 2017). Autophagy plays a key role in cellular homeostasis maintenance and tumorigenesis. A relevant study has indicated that in the progress of affecting lipid metabolism in hypoxic environments, autophagy could maintain the proliferation of HCC cells and promote cancer cell survival (Toshima et al., 2014). It is worth noting that the metabolism-related pathways were closely linked with patients in the low-risk groups. Many studies illustrate the role of metabolic-related pathways in HCC progression; for instance, CD147, which is overexpressed in many cancers, influences tumor progression by promoting the reprogramming of fatty acid metabolism (Li et al., 2015). These results suggested that m5C-related lncRNAs may participate in the genesis and development of HCC by the pathways mentioned above, but further experimentation verification is needed. LncRNAs are known to be expressed in various immune cells and play a vital role in controlling the development and differentiation of these immune cells (Atianand et al., 2017). Tumor infiltration of immune cells in TME, which influences the prognosis of many tumor patients, is attracting much attention. In this study, we made an in-depth analysis of the relationship between risk scores and tumor-infiltrating immune cells using seven common methods. We found higher infiltrating levels of Treg cells, CD4 + T cells, neutrophils, M1 macrophages, and M2 macrophages in the high-risk group than in the low-risk group. Alternatively, endothelial cells and hematopoietic stem cells had a higher expression level in the low-risk group. Based on previous studies, the increased expression of tumor-associated neutrophils, M2 macrophages, and Treg cells are correlated with adverse clinical outcomes in HCC patients (Zhou et al., 2016; Wu et al., 2021). Our results were consistent with previous results. Moreover, the increased activities of type II IFN response meant that tumor immune surveillance and elimination play a role in the high-risk group (Kaplan et al., 1998; Liang et al., 2022). Immunotherapy has received much attention and is expected to become a promising therapeutic method in HCC. We used TIDE algorithm to evaluate the immunotherapeutic response. The result indicated that HCC patients in the high-risk group had a better response to immunotherapy.

ICB therapy, such as anti-PD-L1 antibodies, has shown good prospects in a variety of malignancies. In HCC, the anti-PD-1 antibodies and the anti-Cytotoxic T-Lymphocyte Antigen 4 (CTLA-4) antibodies have been approved for second-line treatment (Pinter et al., 2021). However, immune-related adverse events occur during therapy. Thus, predictive biomarkers for ICB response are urgently needed to maximize the efficacy and keep more patients from adverse effects and heavy economic burden of immunotherapy. Therefore, we compared the expression level of 34 immune checkpoint genes and found a higher expression in the high-risk group. The results above prove that the risk model could predict the expression level of immune checkpoints and is expected to provide important insights into the enhancement of immunotherapy efficacy. Recent studies have found that tumor mutation burden was related to the production of antitumor neoantigens and was identified as a useful biomarker to predict the response to immunotherapy, especially PD-L1 therapy (Chan et al., 2019). As shown in our result, TMB was higher in the high-risk group than the low-risk group, indicating better sensitivity to immunotherapy in the high-risk group. Furthermore, survival analysis suggested that patients with a high burden of tumor mutations had poor prognoses than patients with a low burden. Besides, we combined TMB and risk score and analyzed their survival. The prognosis of patients with high tumor mutation loads in the high-risk subgroup was the worst. Taken together, our research is the first study to explore the relationship between m5C-related lncRNA prognostic risk model and immune cell infiltration, especially immunotherapy.

Tumor resistance to chemotherapeutic drugs often makes chemotherapy unsatisfactory, and thus, efficient and individualized drugs and targets are needed to benefit more HCC patients (Wu et al., 2021). Drug sensitivity analysis suggested that doxorubicin, gemcitabine, and mitomycin are ideal choices for HCC patients in the high-risk group, while axitinib, dasatinib, erlotinib, sorafenib, and rapamycin are suitable for patients in the low-risk group. We also explored the therapeutic potential of five m5C-related lncRNAs by analyzing their association with drug sensitivity of some small-molecule drugs. Our results showed that ELFN1-AS1 was sensitive to dromostanolone propionate, vorinostat, denileukin diftitox ontak, and vismodegib. NRAV was sensitive to vandetanib, dacomitinib, afatinib, lbrutinib, copanlisib, and erlotinib. Ibrutinib is a first-in-class oral irreversible inhibitor of BTK (Bruton’s tyrosine kinase) and has been demonstrated to be an effective treatment for chronic lymphocytic leukemia and other B-cell lymphomas (Ahn and Brown, 2021). Erlotinib, an epidermal growth factor receptor tyrosine kinase inhibitor, is used to treat some types of non-small cell lung cancer and advanced pancreatic cancer (Carter et al., 2022). Vorinostat (Lin et al., 2021), dacomitinib (Ji et al., 2021), vandetanib (Carvalho et al., 2022), afatinib (Wu et al., 2021), and vismodegib (Duplaine et al., 2021) also have anticancer effects in malignancies. In the future, further experiments are required to confirm their therapeutic potential for the targeted therapy of HCC.

However, there are some shortcomings and limitations in our study. For example, we constructed and validated our m5C-related lncRNA risk model using TCGA database, lacking external validation from ICGC or GEO databases for lack of expression data of some selected m5C-related lncRNAs. In addition, we validated the five m5C-lncRNA expression levels using RT-qPCR, but further underlying molecular mechanisms studies are required to make the prediction results more reliable. Moreover, partial clinical information, such as M stage and N stage, was unavailable. Hence, in the future, more clinical and experimental studies are warranted to confirm the accuracy of the prognostic risk model.

## Conclusion

We constructed a new prognostic risk model consisting of five m5C-related lncRNAs. Our risk model proved to be meaningful in functional analysis, immune cell infiltration, tumor mutation load, and drug sensitivity, indicating the prospect of targeting these lncRNAs for improving the responsiveness to immunotherapy and chemotherapy in HCC. To a certain degree, our study provides new insights to support further research on the role of m5C-related lncRNAs in HCC occurrence and development.

## Data Availability

The original contributions presented in the study are included in the article/Supplementary Material, further inquiries can be directed to the corresponding authors.
