# Supplementary material for: Comprehensive analysis of m5C-Related lncRNAs in the prognosis and immune landscape of hepatocellular carcinoma
Source: Front Genet. 2022 Oct 20;13:990594. doi: 10.3389/fgene.2022.990594 (PMC9630339; doi:10.3389/fgene.2022.990594)
Supplement: Supplementary file 1 [file Table4.doc]

Table4. The five m5C-related lncRNA risk model parameters.

| LncRNA | coef |
| --- | --- |
| NRAV | 0.463472818 |
| MKLN1-AS | 0.819925766 |
| AL031985.3 | 0.645159949 |
| ELFN1-AS1 | 0.355295772 |
| AL928654.1 | 0.735015902 |
