# Supplementary material for: Comprehensive analysis of m5C-Related lncRNAs in the prognosis and immune landscape of hepatocellular carcinoma
Source: Front Genet. 2022 Oct 20;13:990594. doi: 10.3389/fgene.2022.990594 (PMC9630339; doi:10.3389/fgene.2022.990594)
Supplement: Supplementary file 2 [file Table6.doc]

Table6. The detail comparison results of correlationship between tumor infiltrating immune cells and risk score.

| Immune | Cor | *p* value |
| --- | --- | --- |
| B cell_TIMER | 0.27819898 | 6.03E-08 |
| T cell CD4+_TIMER | 0.297707148 | 6.20E-09 |
| Neutrophil_TIMER | 0.432110356 | 0 |
| Macrophage_TIMER | 0.41337664 | 1.05E-16 |
| Myeloid dendritic cell_TIMER | 0.368469594 | 3.30E-13 |
| B cell memory_CIBERSORT | 0.136073657 | 0.008773062 |
| B cell plasma_CIBERSORT | 0.117477998 | 0.023826279 |
| T cell CD4+ memory resting_CIBERSORT | -0.164751944 | 0.001472233 |
| T cell CD4+ memory activated_CIBERSORT | 0.179031457 | 0.00054001 |
| T cell follicular helper_CIBERSORT | 0.114569554 | 0.027552962 |
| T cell regulatory (Tregs)_CIBERSORT | 0.213664579 | 3.41E-05 |
| NK cell resting_CIBERSORT | -0.104825047 | 0.043896046 |
| Monocyte_CIBERSORT | -0.146878961 | 0.004639065 |
| Macrophage M0_CIBERSORT | 0.29907014 | 4.41E-09 |
| Neutrophil_CIBERSORT | 0.128241636 | 0.013563078 |
| B cell memory_CIBERSORT-ABS | 0.148632208 | 0.004166735 |
| B cell plasma_CIBERSORT-ABS | 0.164960217 | 0.001451658 |
| T cell CD8+_CIBERSORT-ABS | 0.128713852 | 0.0132196 |
| T cell CD4+ memory activated_CIBERSORT-ABS | 0.181649434 | 0.000445533 |
| T cell follicular helper_CIBERSORT-ABS | 0.248791478 | 1.26E-06 |
| T cell regulatory (Tregs)_CIBERSORT-ABS | 0.347759645 | 5.87E-12 |
| NK cell activated_CIBERSORT-ABS | 0.244288161 | 1.98E-06 |
| Macrophage M0_CIBERSORT-ABS | 0.389406051 | 7.60E-15 |
| Macrophage M1_CIBERSORT-ABS | 0.227887492 | 9.55E-06 |
| Macrophage M2_CIBERSORT-ABS | 0.286567509 | 2.32E-08 |
| Mast cell resting_CIBERSORT-ABS | 0.104936316 | 0.043671209 |
| Neutrophil_CIBERSORT-ABS | 0.172218965 | 0.000879832 |
| B cell_QUANTISEQ | 0.329734744 | 7.83E-11 |
| Macrophage M1_QUANTISEQ | 0.309132807 | 1.24E-09 |
| Macrophage M2_QUANTISEQ | 0.297270426 | 5.51E-09 |
| Monocyte_QUANTISEQ | 0.307903109 | 1.45E-09 |
| T cell CD4+ (non-regulatory)_QUANTISEQ | 0.124221319 | 0.016819672 |
| T cell CD8+_QUANTISEQ | 0.244133328 | 2.01E-06 |
| T cell regulatory (Tregs)_QUANTISEQ | 0.332866428 | 5.05E-11 |
| uncharacterized cell_QUANTISEQ | -0.37729417 | 7.41E-14 |
| T cell_MCPCOUNTER | 0.270236274 | 1.46E-07 |
| T cell CD8+_MCPCOUNTER | 0.118525422 | 0.022595186 |
| cytotoxicity score_MCPCOUNTER | 0.111103451 | 0.032683556 |
| B cell_MCPCOUNTER | 0.219450836 | 2.15E-05 |
| Monocyte_MCPCOUNTER | 0.380689532 | 4.03E-14 |
| Macrophage/Monocyte_MCPCOUNTER | 0.380689532 | 4.03E-14 |
| Myeloid dendritic cell_MCPCOUNTER | 0.194047456 | 0.000177588 |
| Cancer associated fibroblast_MCPCOUNTER | 0.173137268 | 0.000838016 |
| B cell_XCELL | 0.212085537 | 3.91E-05 |
| T cell CD4+ memory_XCELL | 0.303667178 | 2.48E-09 |
| T cell CD4+ central memory_XCELL | -0.113794239 | 0.028627153 |
| T cell CD8+ naive_XCELL | -0.1917927 | 0.000206252 |
| T cell CD8+ central memory_XCELL | -0.115138614 | 0.026786738 |
| Class-switched memory B cell_XCELL | 0.211302099 | 4.18E-05 |
| Common lymphoid progenitor_XCELL | 0.355076829 | 1.96E-12 |
| Common myeloid progenitor_XCELL | -0.139036298 | 0.007397746 |
| Endothelial cell_XCELL | -0.45651362 | 1.90E-20 |
| Granulocyte-monocyte progenitor_XCELL | -0.24852041 | 1.30E-06 |
| Hematopoietic stem cell_XCELL | -0.362891811 | 5.86E-13 |
| Macrophage_XCELL | -0.143063673 | 0.005837584 |
| Macrophage M2_XCELL | -0.285624616 | 2.24E-08 |
| Mast cell_XCELL | 0.23545924 | 4.69E-06 |
| Plasmacytoid dendritic cell_XCELL | -0.146237722 | 0.004823531 |
| B cell plasma_XCELL | -0.12681151 | 0.014651678 |
| T cell CD4+ Th2_XCELL | 0.414180105 | 9.07E-17 |
| stroma score_XCELL | -0.40106336 | 5.31E-17 |
| microenvironment score_XCELL | -0.225919128 | 1.21E-05 |
| Cancer associated fibroblast_EPIC | 0.309687809 | 1.41E-09 |
| Macrophage_EPIC | -0.487147578 | 0 |
| uncharacterized cell_EPIC | 0.480555146 | 0 |
