# Supplementary figures and images for: Comprehensive analysis of m5C-Related lncRNAs in the prognosis and immune landscape of hepatocellular carcinoma
Source: Front Genet. 2022 Oct 20;13:990594. doi: 10.3389/fgene.2022.990594 (PMC9630339; doi:10.3389/fgene.2022.990594)

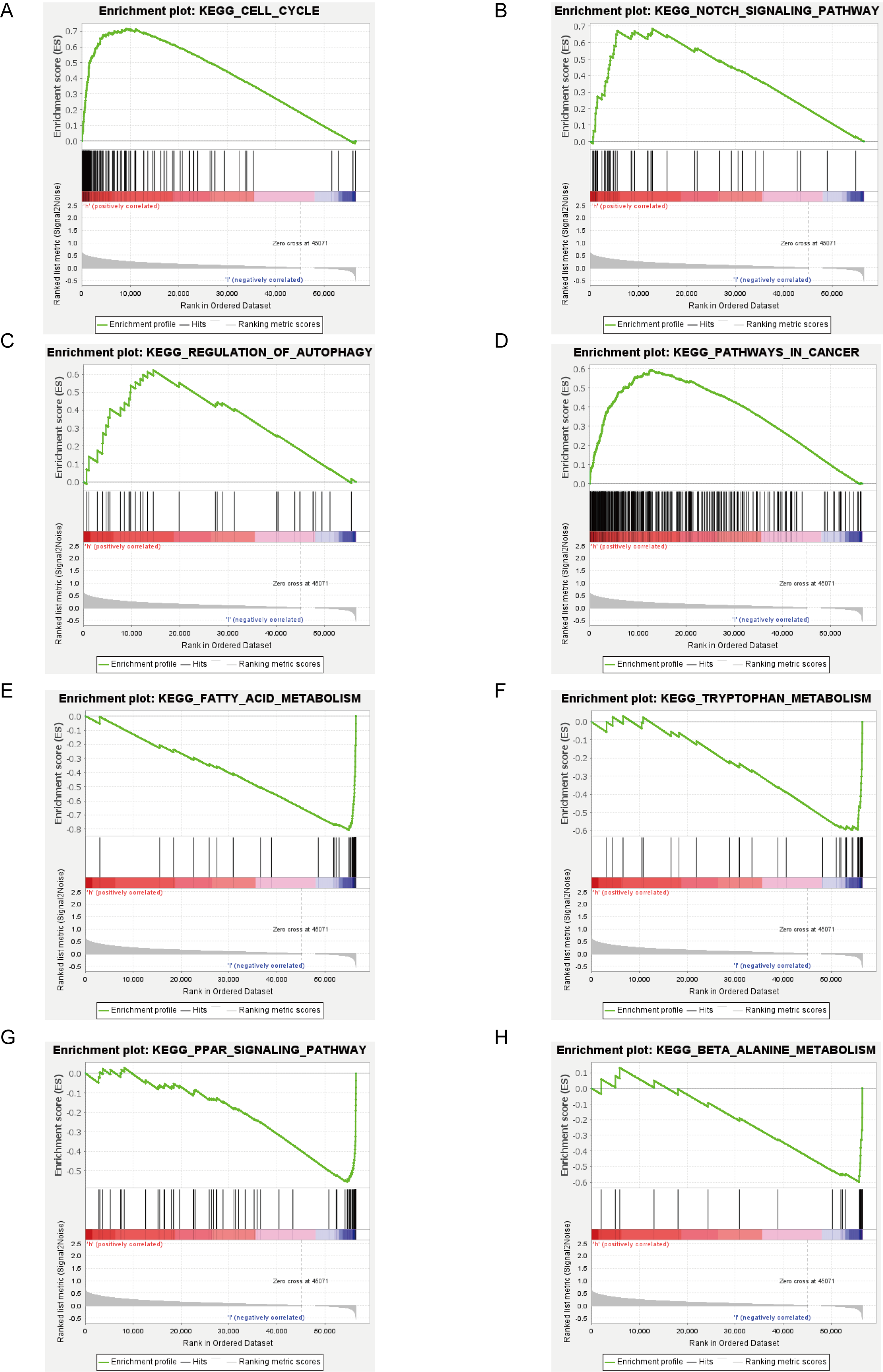

Supplement: Supplementary file 3 [file Image3.tif]

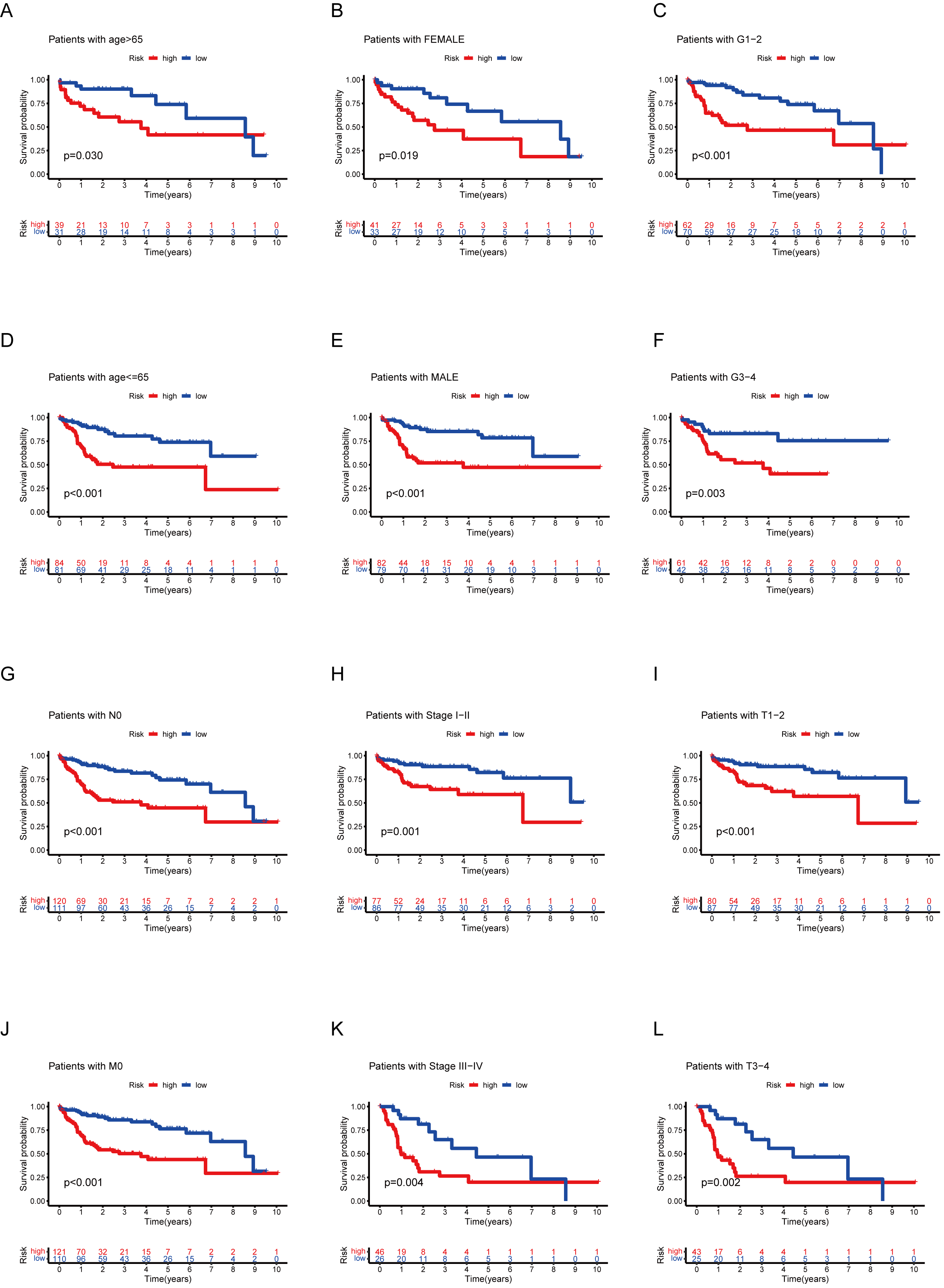

Supplement: Supplementary file 4 [file Image2.tif]

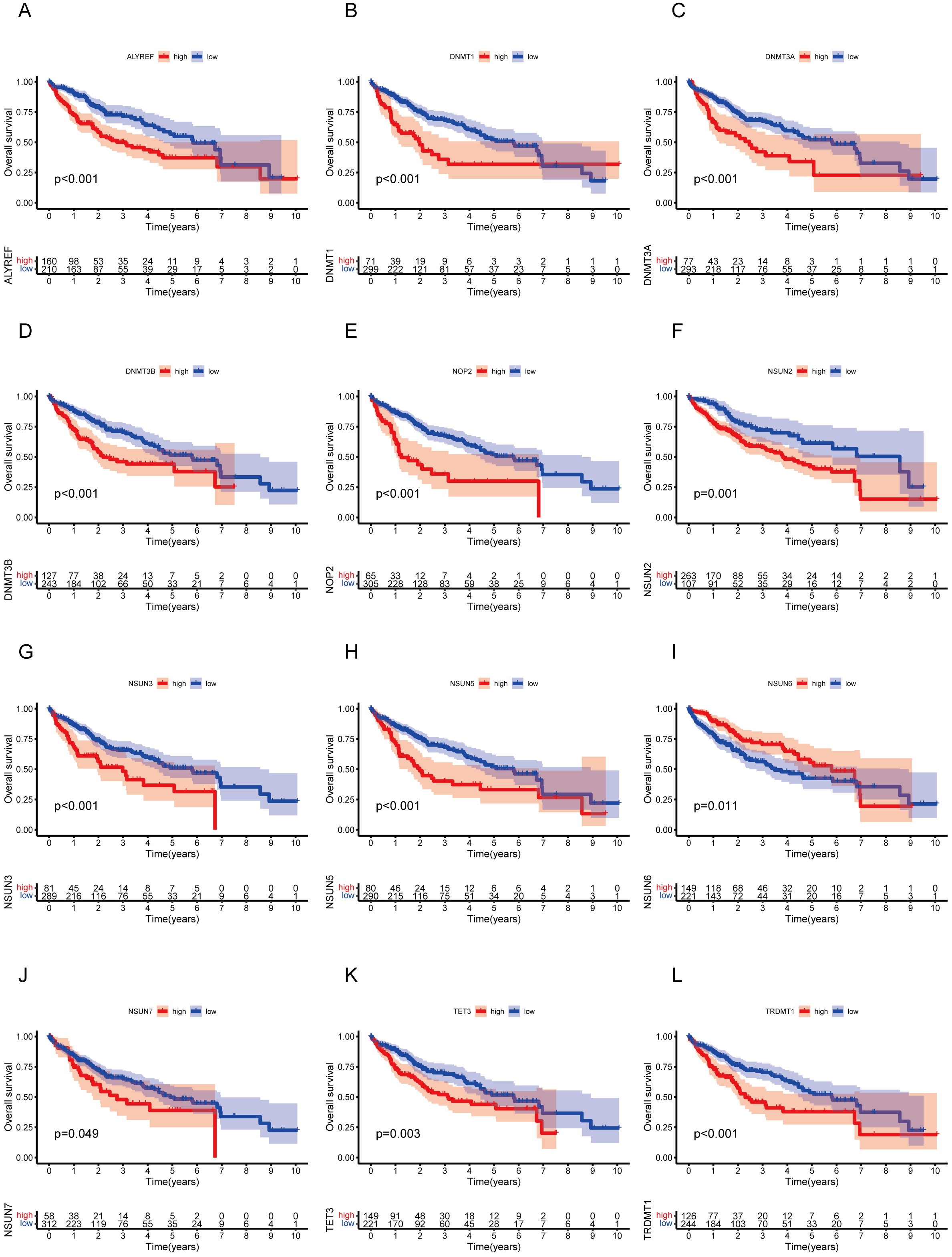

Supplement: Supplementary file 6 [file Image1.tif]
