# Supplementary material for: Comprehensive analysis of m5C-Related lncRNAs in the prognosis and immune landscape of hepatocellular carcinoma
Source: Front Genet. 2022 Oct 20;13:990594. doi: 10.3389/fgene.2022.990594 (PMC9630339; doi:10.3389/fgene.2022.990594)
Supplement: Supplementary file 5 [file Table7.docx]

<https://www.jianguoyun.com/p/DSMhGMAQ1OHYChi8k8gEIAA>
