# Supplementary material for: Comprehensive analysis of m5C-Related lncRNAs in the prognosis and immune landscape of hepatocellular carcinoma
Source: Front Genet. 2022 Oct 20;13:990594. doi: 10.3389/fgene.2022.990594 (PMC9630339; doi:10.3389/fgene.2022.990594)
Supplement: Supplementary file 7 [file Table1.doc]

Table1. The clinical characteristics of patients with hepatocarcinoma in the TCGA database.

| Variables | No. of patients | Percentage (%) |
| --- | --- | --- |
| Age (years) |  |  |
| <=65 | 232 | 62.7 |
| >65 | 138 | 37.3 |
| Gender |  |  |
| Female | 121 | 32.7 |
| Male | 249 | 67.3 |
| Grade |  |  |
| G1 | 55 | 14.86 |
| G2 | 177 | 47.84 |
| G3 | 121 | 32.7 |
| G4 | 12 | 3.24 |
| Unknown | 5 | 1.35 |
| Pathological stage |  |  |
| I | 171 | 46.22 |
| II | 85 | 22.97 |
| III | 85 | 22.97 |
| IV | 5 | 1.35 |
| Unknown | 24 | 6.49 |
| T stage |  |  |
| T1 | 181 | 48.92 |
| T2 | 93 | 25.14 |
| T3 | 80 | 21.62 |
| T4 | 13 | 3.51 |
| Unknown | 3 | 0.81 |
| N stage |  |  |
| N0 | 252 | 68.11 |
| N1 | 4 | 1.08 |
| Unknown | 114 | 30.81 |
| M stage |  |  |
| M0 | 266 | 71.89 |
| M1 | 4 | 1.08 |
| Unknown | 100 | 27.03 |
