# Supplementary material for: Comprehensive analysis of m5C-Related lncRNAs in the prognosis and immune landscape of hepatocellular carcinoma
Source: Front Genet. 2022 Oct 20;13:990594. doi: 10.3389/fgene.2022.990594 (PMC9630339; doi:10.3389/fgene.2022.990594)
Supplement: Supplementary file 8 [file Table5.docx]

Table5. Number of m5C modification sites on prognostic-related lncRNAs.

|  | RNAm5Cfinder (score≥0.1) | m5C-Atlas | iRNAm5C |  |
| --- | --- | --- | --- | --- |
| NRAV | 27 | 2 | 510 |  |
| AL031985.3 | 6 | 0 | 303 |  |
| AL928654.1 | 24 | 0 | 282 |  |
| MKLN1-AS | 21 | 11 | 616 |  |
| ELFN1-AS1 | 14 | 0 | 111 |  |
