# Supplementary material for: Comprehensive analysis of m5C-Related lncRNAs in the prognosis and immune landscape of hepatocellular carcinoma
Source: Front Genet. 2022 Oct 20;13:990594. doi: 10.3389/fgene.2022.990594 (PMC9630339; doi:10.3389/fgene.2022.990594)
Supplement: Supplementary file 9 [file Table3.docx]

| **siRNAs** | |
| --- | --- |
| si-Ctrl sense | UUCUCCGAACGUGUCACGUTT |
| si-Ctrl antisense | ACGUGACACGUUCGGAGAATT |
| si-MKLN1-AS#1 sense | GCCACACUUUGAUCCUAAATT |
| si-MKLN1-AS#1 antisense | UUUAGGAUCAAAGUGUGGCTT |
| si-MKLN1-AS#2 sense | GGGUACUGUCUUGUAGUAUTT |
| si-MKLN1-AS#2 antisense | AUACUACAAGACAGUACCCTT |

Table3. The sequences of siRNAs used in this study.
