# Supplementary material for: Comprehensive analysis of m5C-Related lncRNAs in the prognosis and immune landscape of hepatocellular carcinoma
Source: Front Genet. 2022 Oct 20;13:990594. doi: 10.3389/fgene.2022.990594 (PMC9630339; doi:10.3389/fgene.2022.990594)
Supplement: Supplementary file 10 [file Table2.doc]

Table2. The primers sequences used for qRT-PCR.

| Primer Name | Primer Sequence | |
| --- | --- | --- |
| NRAV | NRAV-F  NRAV-R | AGACCACAATGCACCGCTC  ACTGCAGTACAGTCCTTGGC |
| AL031985.3 | AL031985.3-F  AL031985.3-R | TGTGGTCCCTGTCACACCTA  AGAAGCCAAGGATTCCCCTA |
| AL928654.1 | AL928654.1-F  AL928654.1-R | TGGTAGATCCTCCCCTGGTG  GCTCCATGTCCTCATGCTCT |
| MKLN1-AS | MKLN1-AS-F  MKLN1-AS-R | CCGGGCCAATGTCCTATCTC  AAGCGCTTACACCTCAGACC |
| ELFN1-AS1 | ELFN1-AS1-F  ELFN1-AS1-R | ACCCAAAGTCAAGCTGACCC  CGGCGTCAACTTTTGGTGTA |
